# Comparative evaluation of SNVs, indels, and structural variations detected with short- and long-read sequencing data

**DOI:** 10.1038/s41439-024-00276-x

**Published:** 2024-04-17

**Authors:** Shunichi Kosugi, Chikashi Terao

**Affiliations:** 1 Center for Genome Informatics, Research Organization of Information and Systems, Joint Support-Center for Data Science Research, Shizuoka, Japan; 2 Advanced Genomics Center, National Institute of Genetics, Shizuoka, Japan; 3 Laboratory for Statistical and Translational Genetics, RIKEN Center for Integrative Medical Sciences, Yokohama, Kanagawa Japan; 4 Clinical Research Center, Shizuoka General Hospital, Shizuoka, Japan; 5 The Department of Applied Genetics, The School of Pharmaceutical Sciences, University of Shizuoka, Shizuoka, Japan

**Keywords:** Genome informatics, Genomic analysis

## Abstract

Short- and long-read sequencing technologies are routinely used to detect DNA variants, including SNVs, indels, and structural variations (SVs). However, the differences in the quality and quantity of variants detected between short- and long-read data are not fully understood. In this study, we comprehensively evaluated the variant calling performance of short- and long-read-based SNV, indel, and SV detection algorithms (6 for SNVs, 12 for indels, and 13 for SVs) using a novel evaluation framework incorporating manual visual inspection. The results showed that indel-insertion calls greater than 10 bp were poorly detected by short-read-based detection algorithms compared to long-read-based algorithms; however, the recall and precision of SNV and indel-deletion detection were similar between short- and long-read data. The recall of SV detection with short-read-based algorithms was significantly lower in repetitive regions, especially for small- to intermediate-sized SVs, than that detected with long-read-based algorithms. In contrast, the recall and precision of SV detection in nonrepetitive regions were similar between short- and long-read data. These findings suggest the need for refined strategies, such as incorporating multiple variant detection algorithms, to generate a more complete set of variants using short-read data.

## Introduction

Genomic variants found by comparison with reference genomes (GRCh37, GRCh38, and T2T-CHM13 in humans) are classified into three classes according to size: single nucleotide variant (SNV), short insertion and deletion of less than 50 bp (indel), and structural variation of 50 bp or more (SV). Genomic variants can be detected in a high-throughput manner using whole-genome sequencing (WGS) data consisting of 100–300 bp short reads. Accurately and efficiently detecting variants is important since it is essential for a variety of genetic, clinical, and evolutionary analyses. However, short reads often produce incorrect alignments to repetitive regions, including simple tandem repeats (STRs)^1^ and segmental duplications (SegDups)^2^, which cause erroneous variant calls^3^. Since the maximum allowable insertions and deletions within a read are approximately 15% of the read length for short read alignment tools^4,5^, computational methods to detect large variants (>10 bp short indels and SVs) commonly use indirect alignment signals such as split reads, read pairs, read depth, and local assemblies^6,7^. Thus, it is difficult to accurately and effectively detect large variants using short reads.

Third-generation sequencing technology, offered by Pacific Bioscience (PacBio) and Oxford Nanopore Technology (ONT) to generate long reads with typical lengths of 10 to 30 kb, has recently advanced to generate long reads with >99.9% accuracy for PacBio HiFi and >98% accuracy for ONT long reads. Long reads span many repetitive regions and variants, allowing for effective variant detection^8^. The Genome in a Bottle Consortium (GIAB) developed a benchmark set of integrated variant call sets from short- and long-read WGS data for several human individuals^9–12^. In this project, long reads improved variant calls, especially in repetitive regions containing segmental duplications and regions with low mappability of short reads^9,11^. Fully phased genome assemblies using long-read WGS of 35 individuals identified a significant number of variants that were not observed in short-read WGS data^13^. PacBio HiFi long reads identified more de novo indels and SVs with greater accuracy than short reads^14^. However, short-read data have been routinely used for variant detection, especially in multiple samples, due to the high cost of long-read sequencing and the high demands on the quality and quantity of input DNA.

Many computational algorithms have been developed to detect many variants using short- and long-read sequencing data. Recently, deep learning methods such as DeepVariant^15^, NanoCaller^16^, and PEPPER-Mergin-DeepVariant^17^ have been employed to detect SNVs and indels in a haplotype-aware manner. The accuracy and variability of variants detected depend largely on variant detection algorithms rather than on read alignment tools, library preparation, or sequencing platforms^18,19^. Therefore, knowing the differences in variant detection algorithms using short- and long-read data and the limitations of short read-based variant detection algorithms is important. Many studies have evaluated the precision and recall of variant calling algorithms for SNVs/indels^19–30^ and SVs^31–34^. However, few studies have comprehensively evaluated SNV, indel, and SV detection algorithm performances using short- and long-read WGS data to determine the differences in variants detected in short- and long-read data. To our knowledge, only one study has evaluated SNV/indel detection algorithms, such as GATK^35^, DeepVariant, and Sentieon, for germline variants using both short- and long-read data^29^.

In this study, we evaluated the performance (precision and recall) of a total of 21 popular variant detection algorithms using short- and long-read WGS datasets of NA12878 and HG002 individuals. The variant call set, including SNVs, indels, and SVs, detected from each algorithm was divided into nonrepetitive and repetitive regions containing STR and SegDup, and the performance differences between short and long reads were compared. The results indicate that long reads are more accurate and sensitive than short reads for detecting indels and SVs in repetitive regions, whereas significant variation exists in the performance of short read-based variant detection algorithms. In contrast, the difference between short and long leads was not as great in the nonrepetitive region as that in the repetitive region. Furthermore, detecting indels, especially insertions, by short read-based algorithms became less sensitive as insertions increased in size, especially in the 10−50 bp range, suggesting that indel calling using short reads needs to cover indels of this size.

## Methods

### WGS datasets

A summary of the WGS dataset used in this study is presented in Supplementary Table S1. The NA12878 and HG002 short-read WGS datasets were Illumina 150 bp and 148 bp paired-end reads with 36.7× and 30× coverage, respectively. The long-read WGS datasets of NA12878 and HG002 included PacBio HiFi/CCS reads with 29.2× and 39.9× coverage and 10.0 kb and 19.1 kb N50 read lengths, respectively (Supplementary Table S1). All reads were obtained from the European Nucleotide Archive (ENA, https://www.ebi.ac.uk/ena/browser/home). All short and long reads were aligned to GRCh37 (hs37d5) using bwa mem (v0.7.17, https://github.com/lh3/bwa) for short reads and Minimap2 (v2.24)^36^ with -ax map-hifi –MD options for long reads.

### Reference variant datasets

The reference variant dataset of all variant types for NA12878 and the SNV/indel reference variant dataset for HG002 were based on long read-based haplotype-resolved HGSVC variant data (variants_freeze4_snv_snv_alt.vcf.gz, variants_freeze4_indel_insdel_alt.vcf.gz, and variants_freeze4_sv_insdel_alt.vcf.gz)^13^, which were obtained from http://ftp.1000genomes.ebi.ac.uk/vol1/ftp/data_collections/HGSVC2/release/v2.0/integrated_callset/. Variants corresponding to NA12878 and HG002 were extracted from each vcf file. The coordinates of these variants were converted to GRCh37 coordinates using liftOver with the hg38ToHg19.over.chain file (downloaded at UCSC: https://genome.ucsc.edu). The GIAB benchmark SNVs (v4.2.1) for NA12878 and HG002, which were obtained from https://ftp-trace.ncbi.nlm.nih.gov/ReferenceSamples/giab/release/, were merged with the HGSVC2 dataset without redundancy, resulting in 69,838 and 77,963 additional SNVs for NA12878 and HG002, respectively. For HG002 indels, the GIAB indel set was merged with the HGSVC indel set without redundancy, resulting in 65,646 additional indels. In addition, the 1KGP variant datasets created from the 1KGP high-coverage WGS data were obtained from http://ftp.1000genomes.ebi.ac.uk/vol1/ftp/data_collections/1000G_2504_high_coverage/working/20220422_3202_phased_SNV_INDEL_SV/, and the SNVs and indels corresponding to NA12878 were extracted. The coordinates of the extracted NA12878 SNVs/indels were converted to GRCh37 coordinates and merged with the NA12878 SNV and indel reference datasets without redundancy, resulting in 70,437 and 93,764 additional SNVs and indels, respectively. High-confidence indels from the PEPPER indel calls detected using PacBio HiFi long-read WGS data for NA12878 and HG002 were merged with their respective indel reference data without redundancy, resulting in 143,368 and 144,515 indels for NA12878 and HG002, respectively. The high-confidence indels from the PEPPER calls were variants at sites with a single nonreference allele, and 200 indels randomly selected from these indels showed nearly 100% precision, as verified by manual visual inspection with the IGV viewer (https://igv.org). For SVs of HG002, the GIAB Tier1 v0.6 benchmarked SV sets were obtained from ftp://ftp-trace.ncbi.nlm.nih.gov//ReferenceSamples/giab/release/AshkenazimTrio/HG002_NA24385_son/NIST_SV_v0.6/, and <50 bp SVs were removed. To add SVs with high confidence to the reference datasets, eight long-read-based SV detection algorithms (cuteSV, dysgu, NanoVar, pbsv, Sniffles, SVDSS, SVIM, and TRsv, the last of which is unpublished algorithms; see Table 1) were used to select high-confidence SVs that were commonly detected by at least four algorithms in the SV call sets created from HiFi long-read WGS data of NA12878 or HG002. All of the long read-based tools tested in this study were included in the tools used to generate the high-confidence SV set. This approach was fair for all testing tools and minimized potential evaluation bias. Overlapping SV selection was based on breakpoint distances of ≦200 bp for INS and ≧50% reciprocal overlap for the other types. The selected high-confidence SVs for NA12878 and HG002 were merged without redundancy with the HGSVC reference SV and GIAB SV sets, resulting in 6653 and 24,052 additional SVs, respectively. All the reference indels and SVs included only ≦50 bp and ≧50 bp variants, respectively. The final reference SNVs, indels, and SVs for NA12878 and HG002, including those in the STR and SegDup repeat regions, are summarized in Supplementary Table S2. Overlapping variants between STR and SegDup were considered STR-overlapping variants.

**Table 1:** Variant detection algorithms used in this study.

Algorithm	Read type	Variant type	Version	Reference
DeepVariant	short/long	SNV/indel	1.3.0	Poplin et al.^15^
GATK4	short	SNV/indel	4.3.0	DePristo et al.^35^
Lofreq	short	SNV/indel	2.1.5	Wilm et al.^37^
Strelka	short	SNV/indel	2.9.10	Saunders et al.^38^
Platypus	short	indel	1.1.0	Rimmer et al.^22^
NanoCaller	long	SNV/indel	3.4.1	Ahsan et al.^16^
PEPPER^a^	long	SNV/indel	r0.8	Shafin et al.^17^
Manta	short	SV/indel	1.6.0	Chen et al.^40^
DELLY	short	SV	1.1.8	Rausch et al.^41^
GRIDSS	short	SV	2.13.2	Cameron et al.^42^
INSurVeyor	short	SV/indel	1.1.1	Rajaby et al.^43^
Lumpy	short	SV	0.3.1	Layer et al.^44^
Wham	short	SV	1.8.0	Kronenberg et al.^45^
MOPline	short	SV	1.8.2	Kosugi et al.^46^
cuteSV	long	SV/indel	1.0.13	Jiang et al.^47^
Dysgu	short/long	SV/indel	1.3.16	Cleal et al.^39^
pbsv	long	SV/indel	10.2.0	PacBio SMRT Link^b^
Sniffles	long	SV/indel	2.0.2	Sedlazeck et al^48^
SVDSS	long	SV/indel	1.0.5	Denti et al.^49^
SVIM	long	SV/indel	2.0.0	Heller and Vingron^50^

^a^PEPPER: PEPPER-Mergin-DeepVariant.

^b^https://downloads.pacbcloud.com/public/software/installers/smrtlink_12.0.0.177059.zip.

### Variant calling

The variant detection algorithms used in this study with short-read and long-read WGS data are summarized in Table 1. The algorithms were run using Illumina short-read or PacBio HiFi long-read WGS data from NA12878 and HG002. The commands, options, and filtering conditions used for the algorithms are described in the Supplementary Note. For SVs/indels called by long read-based algorithms, only one variant of overlapping variants of the same type at the same or nearly the same position (≦50 bp distance for insertion and ≧50% reciprocal overlap for deletion (DEL) and duplication (DUP)) in the same call set were used for the analysis when the size ratio of the overlapping variants was between 0.67 and 1.5 because of the possibility of making false duplicate calls. Variants inside and outside the repetitive regions (STR and SegDup) were evaluated separately. STR was based on a TRF-based tandem repeat file (simpleRepeat.txt.gz) obtained from UCSC (https://hgdownload.soe.ucsc.edu/goldenPath/hg19/database/) and a HipSTR reference bed file (GRCh37.hipstr_reference.bed.gz) obtained from https://github.com/HipSTR-Tool/HipSTR-references/raw/master/human/. TR regions ranging from 20 to 10,000 bp from both files were used. SegDup was the segmental duplication data (genomicSuperDups.txt.gz) obtained from the UCSC Genome Browser site (https://hgdownload.soe.ucsc.edu/goldenPath/hg19/database/). The total length of STR regions without overlap was approximately 71.1 Mb. The total length of SegDup without overlap was approximately 103 Mb. The total overlapping length between the STR regions and the SegDup regions was approximately 7 Mb. Overlapping variants between STR and SegDup were considered STR-overlapping variants.

### Evaluation of variant detection algorithms

The variant calls of various variant detection algorithms were evaluated using the reference variant sets of the corresponding variant type and sample. The SNV calls that matched the reference SNV position and nonreference allelic base of NA12878 or HG002 were determined to be true positive (TP) calls. For indel calls, when the reference indel was located within 0.5 times the size of the called indel and the ratio of the called to the matched reference indel size was between 0.5 and 2.0, the called indel was considered TP. As an exception for 1-bp indels, when the distance between the called and reference indel positions was 1 bp, the called indel was considered TP. SV calls were considered TPs when they met the following criteria: the breakpoint distance between the called insertion (INS) and the reference INS was ≤200 bp, and the overlap length between the called DEL and the reference DEL was ≥50% of the respective length (≥ 50% reciprocal overlap). For long-read-based algorithms, the ratio of the called INS size to the matched reference INS size had to be between 0.5 and 2.0. DUP calls were considered INS calls since DUP is a type of INS and is either not called or called infrequently in many long read-based algorithms. When the distance between the breakpoints of the DUP call and the reference INS was within 200 bp and the ratio between the called DUP size and the matched reference INS size was 0.5 to 2.0, the DUP call was considered TP for INS.

The reference variant sets created lacked true variants that have yet to be found. Therefore, we reevaluated the putative false positive (FP) calls that did not match the reference variants by manual visual inspection of long-read alignments. To do this, we randomly selected 50 variants from the initial tentative FP calls in each nonrepetitive and repetitive region and for each variant type (ins, del, INS, and DEL). For indels, 50 variants were randomly selected from each of two size ranges (1−5 bp and 6−50 bp) for insertion and deletion, respectively. The selected variants were validated by manually observing evidence supporting the presence of the variant in the long-read alignment using the IGV viewer and the corresponding PacBio HiFi long-read bam file. The criteria for determining TP were the same as those for the reference-based evaluation, and variant calls with at least two long reads that met the criteria were considered TP. For INS calls from short read-based algorithms, when the size of the INS observed in the long-read alignments was <10 bp, the call was considered an FP. For <100 bp DUP calls, when the size of INS observed in the long-read alignment was between 30 − 200 bp, the call was considered TP. For >500 bp DUP calls, when the size of INS observed in the long-read alignment was greater than 0.8-fold the size of the DUP call, the call was considered TP. For INS and DUP calls, when there were long read alignments with at least two 5’-clipped ends around the first breakpoint of the variant and at least two 3’-clipped ends around the second breakpoint of the variant (the first breakpoint for INSs), the call was considered TP. Eventually, the true positive rate in the initial FP calls was estimated with the number of TPs observed in the validated variants. The precision (*Pr*), recall (*Rc*), and F-measure (*F*) were calculated as follows:

$$\Pr =\frac{{TP}1+{TP}2}{{Call}}\times 100$$

$${Rc}=\frac{{TP}1+{TP}2}{{Ref}}\times 100$$

$$F=\frac{\Pr \times {Rc}\times 2\times 0.01}{\Pr +{Rc}}$$

where *TP1*, *TP2*, *Call*, and *Ref* are the number of true positives that matched the reference, the estimated number of true positives among the initial FPs that did not match the reference, the number of called variants, and the number of corresponding reference variants, respectively. The number of reference variants for each variant type changed to the maximum number of TP calls from an algorithm if the number of TP calls exceeded the number of corresponding reference variants. Because our manual visual inspection starategy tests for 50 variants randomly selected from the initial FP calls for each variant type and estimates the final precision and recall, the estimates are subject to error. The binomial test using the precision values determined in the visual inspection tests of 50 variants was used to determine confidence intervals for the estimated precision of the initial FP calls. The confidence intervals for the final precision and recall were determined using the number of initial TP calls and the determined confidence intervals.

## Results

### Datasets and strategy for evaluating variant detection performance

We used NA12878 and HG002 benchmarked human datasets, Illumina short-read WGS data and PacBio HiFi long-read WGS data to evaluate the performance of variant detection algorithms. Variants detected from haplotype-resolved assemblies^13^ were used for all variant types as the reference variant datasets for NA12878 and HG002 (Supplementary Table S2). For the SNV and indel reference datasets, the GIAB benchmark SNVs and indels (v4.2.1) and the 1000 Genomes Project (1KGP) SNVs (only for NA12878) were integrated without redundancy. Although the benchmarked variant datasets from GIAB and haplotype-resolved long-read assemblies cover high-quality variants, many variants were missed, especially in repetitive regions, such as STRs. Hence, we further merged the high-confidence indels and SVs from HiFi long reads with the indel and SV reference datasets (see Methods for details). Variant calls that matched or overlapped the reference variants were considered TP, and variant calls that did not match the reference variants were considered tentative false positives (tFPs). We expected that the tFP calls included some of the TP calls that were missed in the reference variant sets. To estimate the TP content in tFPs, 100 variants (200 variants in different size ranges of indels) randomly sampled from tFP calls of each variant type were further validated by manual visual inspection using the IGV viewer (see Methods for details). Precision and recall were calculated by combining the estimated TP calls in tFP with the initial TP calls determined using the reference variant set, and they were determined for variants inside and outside repetitive regions separately.

### SNVs can be detected with similar precision and recall levels for both short- and long-read data

**Fig. 1:**
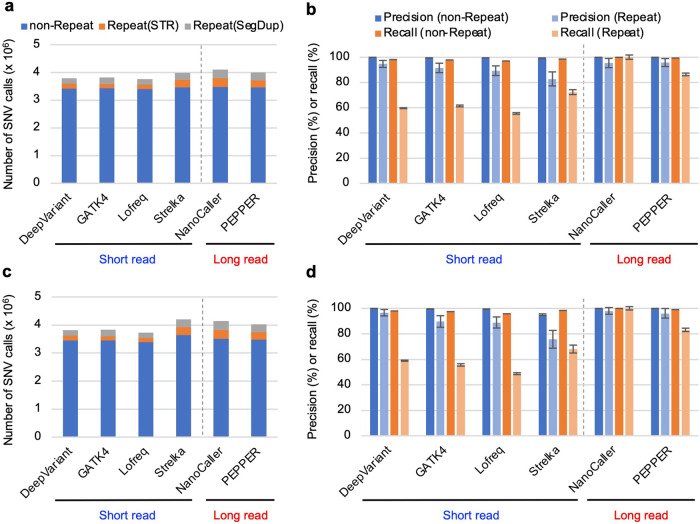
Evaluation of SNVs called with short and long read-based SNV detection algorithms. **a** Number of SNV calls for NA12878. SNVs were detected with the indicated short read-based (DeepVariant, GATK4, Lofreq, and Strelka) and long read-based (NanoCaller and PEPPER/DeepVariant) SNV detection algorithms using NA12878 Illumina short read or PacBio HiFi long read WGS data. The blue, orange, and gray bars indicate the SNV calls present in nonrepetitive (nonrepeat), STR (Repeat(STR)), and segmental duplication (Repeat(SegDup)) regions, respectively. **b** Precision and recall of SNV calls for NA12878. The SNV calls for each algorithm were evaluated with the NA12878 reference SNVs and by manual visual inspection. The blue and light blue bars indicate the precision values of SNVs present in nonrepetitive and repetitive regions, respectively. Orange and light orange bars indicate the recall values of SNVs present in nonrepetitive and repetitive regions, respectively. The confidence interval with each bar is based on the estimated errors from the manual visual inspection of 50 variants from the initial FP calls. **c** Number of SNV calls for HG002. Bars are represented in **a**. **d** Precision and recall of SNV calls for HG002. Bars are represented as in **b**.

To evaluate SNV calling for NA12878 and HG002, we selected four popular short-read-based algorithms (DeepVariant^15^, GATK4^35^, Lofreq^37^, and Strelka^38^) and two long-read-based algorithms (NanoCaller^16^ and PEPPER-Mergin-Deepvariant^17^, the latter is hereafter referred to as PEPPER), among which DeepVariant, NanoCaller, and PEPPER are deep learning-based algorithms. The recall values (50–70%) of short-read-based SNV detection algorithms in repetitive regions (STRs and SegDups) were lower than those (83–100%) of long-read-based detection algorithms. However, the precision and recall in nonrepetitive regions were comparable between short- and long-read data (Fig. 1 and Supplementary Table 3). The best algorithms for SNV detection were DeepVariant for short reads and NanoCaller for long reads. The F-measure scores across the genome were similar between the short- and long-read-based algorithms since the number of SNV calls in the repeat region was only 15% of the total SNV calls. This suggests that SNV calls using short-read WGS data can be expected to be as reliable as those using long-read data.

### Low recall of short insertions in short-read data

**Fig. 2:**
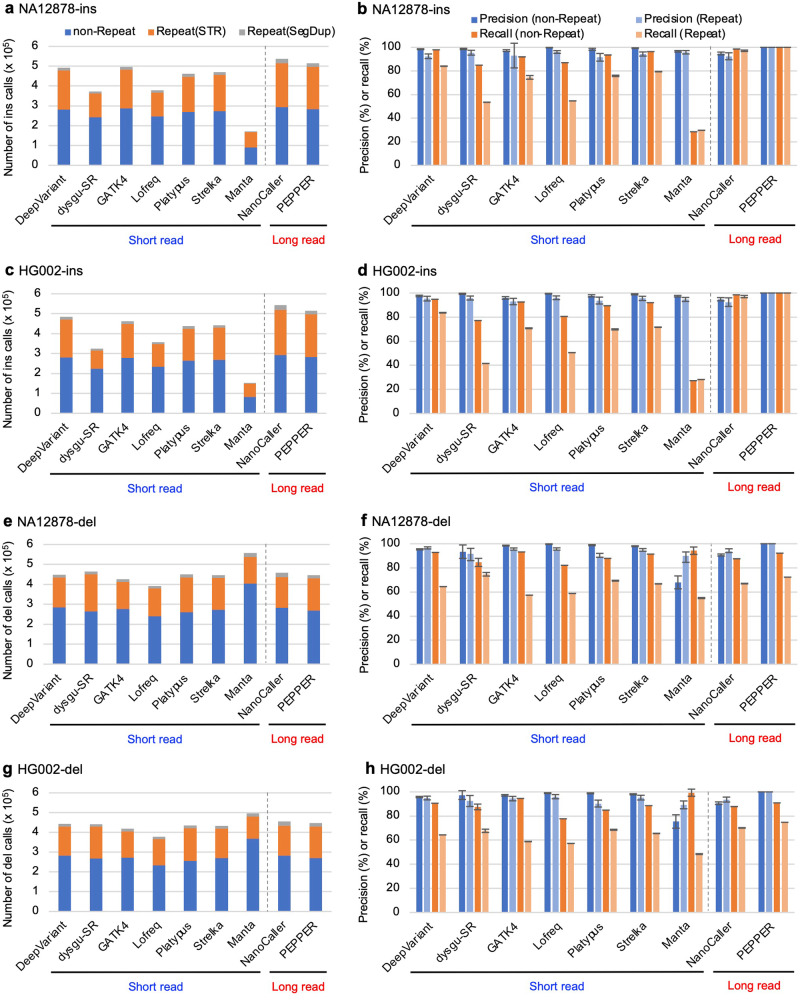
Evaluation of indels called with short read-based and long read-based indel detection algorithms. **a** Number of insertion (ins) calls for NA12878. Insertions in the 1–50 bp size range were detected with the indicated short read-based (DeepVariant, dysgu-SR, GATK4, Lofreq, Platypus, Strelka, and Manta) and long read-based (NanoCaller and PEPPER/DeepVariant) indel detection algorithms using NA12878 Illumina short read or PacBio HiFi long read WGS data. Bars are represented as in Fig. 1. **b** Precision and recall of ins calls for NA12878. Indel calls for each algorithm were evaluated with the NA12878 reference indels and by manual visual inspection. Bars are represented in Fig. 1. **c** Number of ins calls for HG002. **d** Precision and recall of ins calls for HG002. **e** Number of deletion (del) calls for NA12878. Deletions in the 1–50 bp size range were detected with the indicated tools. **f** Precision and recall of del calls for NA12878. **g** Number of del calls for H002. **h** Precision and recall of del calls for HG002.

Three algorithms (dysgu^39^, Platypus^22^, and Manta^40^) were selected to evaluate short indels in addition to the six SNV detection algorithms in the previous sections that simultaneously detect indels and SNVs. We used dysgu (dysgu-SR) with short-read WGS data to evaluate its ability to call indels because dysgu detects indels and SVs with short- or long-read WGS data. Manta used short-read WGS data to detect not only SVs but also short indels less than 50 bp, depending on the parameter settings. Platypus uses a combination of local alignment and local assembly of short reads to detect indels. The 1–50 bp indel calls from each algorithm were evaluated separately for insertion (ins) and deletion (del). Approximately 50% of the indels were derived from repetitive regions, and more than 90% of the indels in repetitive regions were in STRs, even though STRs represent only 2.6% of the human genome (Fig. 2). We detected differences in the calling result characteristics between ins and del. The recall values of short-read-based ins calls in repetitive regions were significantly lower than those of long-read-based calls, regardless of the algorithm used. Long-read-based PEPPER showed almost 100% precision and recall even in repetitive regions (together with nonrepetitive regions). DeepVariant was the best short read-based algorithm for ins calling in repetitive regions, achieving > 80% recall. GATK4, Platypus, and Strelka also performed well for ins calling. Unexpectedly, the recall values for dels in repetitive regions were comparable between short- and long-read-based algorithms and were considerably lower than those in nonrepetitive regions for both short- and long-read-based algorithms (Fig. 2). All of the short read-based algorithms performed similarly in del calling, and the long read-based PEPPER achieved nearly 100% precision (but not recall) in del calling in both repetitive and nonrepetitive regions.

### Large indel insertion detection is difficult with short-read data

**Fig. 3:**
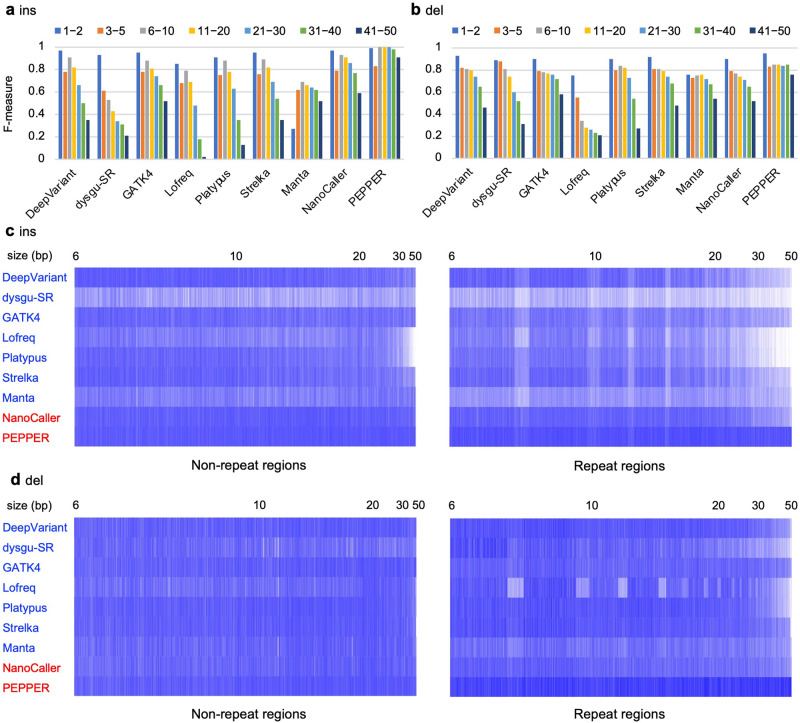
Evaluation of insertion calls by size in NA12878. **a** Accuracy for indel insertion calls across sizes in NA12878. F-measures of insertion calls for the indicated short read-based and long read-based algorithms are shown across size ranges with color bars (blue: 1 and 2 bp, orange: 3−5 bp, gray: 6−10 bp, yellow: 11−20 bp, cyan: 21−30 bp, green: 31−40 bp, black: 41−50 bp). **b** Accuracy for indel-deletion calls across sizes in NA12878. The F-measures of the deletion calls for the indicated algorithms are shown in **a**. **c** The distribution of 6−50 bp in the TP calls matched that of the NA12878 reference across sizes. For NA12878 insertions called with the indicated short read-based (marked with blue letters) and long read-based (marked with red letters) indel detection algorithms, the reference insertions (i.e., TP calls) that matched with the insertion calls are plotted with blue bars. The reference insertions ordered by size are indicated on the x-axis, with representative sizes at the top. The blanks in white indicate the reference insertions that were not detected by the corresponding algorithms. The left and right panels show the insertions in nonrepetitive and repetitive regions, respectively. The indels shown are restricted to the 6–50 bp range to focus on large indels. **d** Distribution of 6–50 bp deletion TP calls matched with the NA12878 reference across sizes. The plots are represented as in **c**.

We further examined the precision, recall, and F-measure of short indels on a fine scale over a range of seven indel sizes. Overall, the short-read-based algorithms, particularly DeepVariant, Lofreq, and Strelka, were highly accurate across the seven size ranges; however, their recall values for ins decreased as the ins size increased, especially above 10 bp. (Fig. 3, Supplementary Figs. S1–S3). In contrast, the long-read-based algorithms, particularly PEPPER, showed higher values for both precision and recall than short-read-based algorithms across all size ranges in both nonrepetitive and repetitive regions. For del calls, the precision was high for all algorithms in all size ranges. Additionally, the recall of del was comparable between short read-based and long read-based algorithms. However, the recall of del with large size ranges (>30 bp) was low, especially in repetitive regions (Supplementary Figs. S4 and S5). Overall, DeepVariant, GATK4, and Strelka were found to be the best short read-based algorithms for detecting indels, although they were less efficient at detecting large in ss.

Long read-based SV detection algorithms, such as cuteSV, dysgu, Sniffles, and SVIM, can detect short indels depending on parameter settings. To determine whether the indel calling performance of these SV detection algorithms outperforms that of indel calling-specific algorithms, we determined the precision and recall of indel calls from these algorithms using the same datasets. SVIM and dysgu (dysu-LR) showed good precision and recall in detecting ins and del in the 3−50 bp range (Supplementary Figs. S3 and D4), and their performance was greater than that of the short read-based algorithms. The recall of dysgu (dysu-LR) and SVIM in del calling was greater than that of PEPPER, especially in several size ranges and repetitive regions, suggesting that these algorithms can supplement PEPPER in del calling.

### Most SVs in repetitive regions can be detected using only long-read data, but SVs in nonrepetitive regions can be detected with equal efficiency using short- and long-read data

**Fig. 4:**
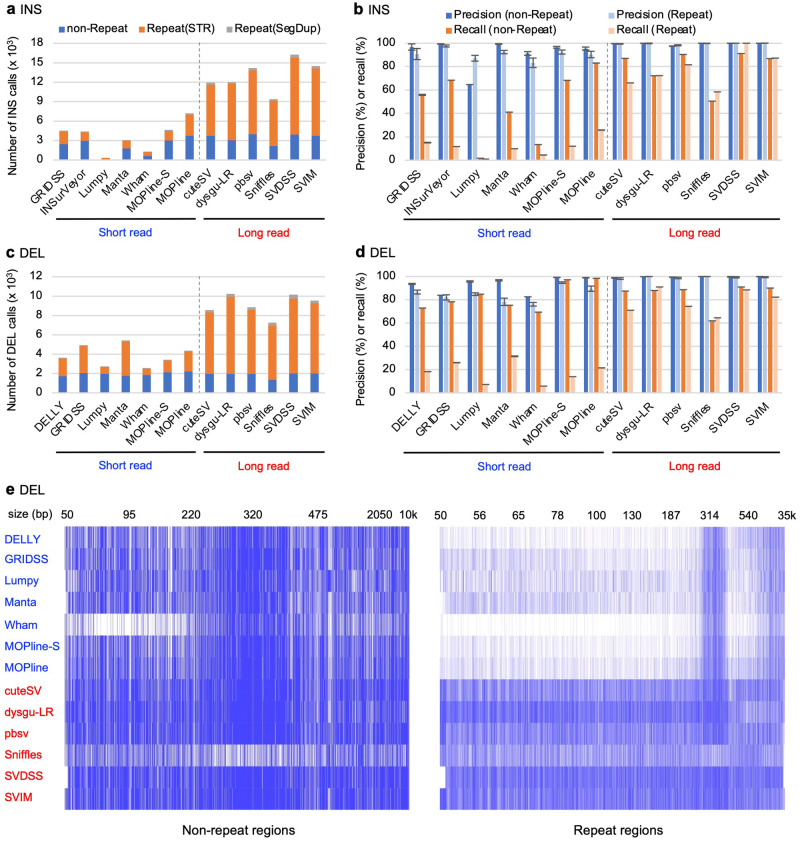
Evaluation of SVs called with short read-based and long read-based SV detection algorithms. **a** Number of insertion (INS) calls for NA12878. INSs and duplications (DUPs) ≥ 50 bp were detected with the indicated short-read-based (GRIDSS, INSurVeyor, Lumpy, Manta, Wham, MOPline-S, and MOPline) and long-read-based (cuteSV, dysugu-LR, pbsv, Sniffles, SVDSS, and SVIM) SV detection algorithms using NA12878 short-read or long-read WGS data. DUP calls were converted to INSs. Bars are represented as in Fig. 1. MOPline-S indicates MOPline-7t in the single sample mode. **b** Precision and recall of INS calls for NA12878. SV calls for each algorithm was evaluated with the NA12878 reference SVs and by manual visual inspection. Bars are represented as in Fig. 1. **c** Number of deletion (DEL) calls with ≥ 50 bp for NA12878. **d** Precision and recall of DEL calls for NA12878. **e** Distribution of DEL TP calls matched with the NA12878 reference across sizes. Among the NA12878 DELs called with the indicated short read-based (marked with blue letters) and long read-based (marked with red letters) SV detection algorithms, the reference DELs (i.e., TP calls) that matched the DEL calls are plotted with blue bars. The reference DELs are ordered by size and are indicated on the x-axis, with representative sizes at the top. The blanks in white indicate the reference DELs that were not detected by the corresponding algorithms. The left and right panels show the DELs in nonrepetitive and repetitive regions, respectively.

We selected DELLY^41^, GRIDSS^42^, INSurVeyor^43^, Lumpy^44^, Manta^40^, Wham^45^, and MOPline^46^ as short-read-based SV detection algorithms and cuteSV^47^, dysgu^39^, pbsv, Sniffles^48^, SVDSS^49^, and SVIM^50^ as long-read-based algorithms to evaluate SV calling performance using short- and long-read data. With the exception of the recently reported INSurVeyor insertion (INS) detection tool, many of these short read-based algorithms have shown good performance in previous studies^31,32^. MOPline is a recently reported ensemble pipeline that selectively combines high-quality SV calls from multiple SV detection algorithms. We used the MOPline-7t algorithm in MOPline, which integrates the results from seven external SV detection tools (CNVnator, GRIDSS, Manta, MATCHCLIP, MELT, inGAP-sv, and Wham) in single sample mode (MOPline-S) and multiple sample mode with the SMC function (MOPline)^46^. Duplication (DUP) calls were converted to INSs since DUPs are a type of INS, and many long read-based algorithms make no or few DUP calls. Approximately 70–80% of INSs and DELs called with long-read data were in repetitive regions (STRs and SegDups). In contrast, 27–58% of INSs and DELs called with short-read data were in repetitive regions (Fig. 4 for NA12878, Supplementary Fig. S8), as observed in a previous study^46^. The recall of INS and DEL calls for short read-based algorithms was significantly lower in repetitive regions than for long read-based algorithms; however, many short read-based algorithms showed a similar level of precision and recall as long read-based algorithms in nonrepetitive regions (Fig. 4, Supplementary Figs. S8 and S9). Despite the obvious advantage of detecting SVs with long-read data, short-read-based algorithms have three features comparable to long-read-based algorithms: (1) comparable precision and recall in nonrepetitive regions, (2) sensitive detection of 300–400 bp DELs that may correspond to Alu deletions, and (3) sensitive detection of large DELs over several kilobases in repetitive regions (Fig. 4, Supplementary Fig. S9). These observations suggest that short-read data can be effectively used to detect SVs for research purposes. Taken together with the results for size-dependent DEL and INS calling efficiency (Fig. 4e, Supplementary Figs. S9 and S10), these findings suggest that the optimal algorithms for detecting SVs are MOPline, Manta, and INSurVeyor for short-read data and SVDSS, SVIM, and pbsv for long-read data. The optimal variant detection algorithms for short and long reads are summarized in Supplementary Table S6.

## Discussion

This study employs a new framework to evaluate variant calling more accurately than traditional strategies using only a set of benchmarked variants. In this framework, a fraction of FP calls determined with the benchmarked reference variant sets are manually visually inspected. The reference variant sets still lack true variants, which results in incorrect FP calls. Therefore, estimating the percentage of TPs in the initial FP calls obtained from the reference sets improves the precision and recall determination. In addition, this strategy can also minimize potential variant bias in the reference variant sets since reference variant sets are often derived from specific tools. However, the estimation is imperfect because only 100–200 variants are visually inspected for each variant type, and the read alignment, even in long reads, is often inaccurate in repetitive areas, which can lead to erroneous determination of true and false calls. Nevertheless, the results of this work should more accurately reflect the actual benchmarks than previous studies that evaluated variant calling algorithms and can faithfully assess the differences in results obtained between short- and long-read data.

Our comprehensive evaluation of SNVs, indels, and SVs, called with many variant detection algorithms, highlights several different or common aspects of the variants detected between short- and long-read data. The obvious difference observed between the short- and long-read data was the lower recall of indel insertions in the short-read data than in the long-read data, with the recall decreasing as the ins size increased. This may be because the efficiency and accuracy of the alignment of short read aligners, such as bwa, to the reference genome are lower for short reads spanning large ins than for those spanning small ins. In contrast to insertions, deletions were detected with a similar level of precision and recall between short- and long-read data. This may be because short-read aligners align del-spanning reads to the genome more efficiently than ins-spanning reads (Supplementary Fig. S11) and because indel detection algorithms detect indirect alignment signals (e.g., split reads and read pairs) for detecting dels more effectively than alignment signals for detecting inss. When long-read data are unavailable, a combination of short-read-based indel detection algorithms with superior performance, such as DeepVariant, GATK4, Strelka, and Manta, may be able to detect indels at a level comparable to that of long-read-based algorithms.

Another striking difference between short- and long-read data was observed for SVs in repetitive regions, particularly in STRs. Short-read-based SV detection algorithms failed to detect both INSs and DELs present in STRs more efficiently than long-read-based algorithms. Many of the INSs, DELs, and short indels present in STRs represent increased or decreased copies of the STR repeat units. Since short read-based SV detection algorithms use only indirect alignment signals to detect SVs, alignments of short reads in STR repeat regions often fail to capture indirect signals to detect increases or decreases in the number of STR repeat units. Short and long read-based SV detection algorithms detected a similar number of SVs with similar precision in nonrepetitive regions and a similar number of large DELs in repetitive regions (see Fig. 4). Thus, several short read-based SV detection algorithms, such as MOPline, Manta, and INSurVeyor, have the potential to cover many SVs that have a significant impact on gene function, even when long read data are not available since many functional SVs are often large SVs and are in nonrepetitive regions, including coding regions. Furthermore, long read-based SV detection also has drawbacks. A previous study showed that SV calls from long-read data often miss large SVs (>10 kb)^46^. This is likely because long read-based SV detection algorithms cannot effectively use read coverage-based or read pair/split read-based methods to detect large SVs.

The SNV recall of short-read-based SNV detection algorithms was lower in repetitive regions than that of long-read-based algorithms: approximately 50–60% of the long-read-based algorithms. However, this difference in efficiency may have little impact on genome-wide SNV detection since only 10–15% of all SNV calls are detected in repetitive regions. DeepVariant, which exhibits a high level of precision and recall comparable to long read-based algorithms in both repetitive and nonrepetitive regions, would be a good candidate for SNV calling with short-read data.

In conclusion, this study revealed that many indels, especially >10 bp insertions, are missed when short-read sequencing data are used. Most SVs in STR regions are also missed when short-read data are used. Thus, improved strategies, such as incorporating multiple variant detection algorithms or alternative algorithms specific to STR variants, are needed to obtain a more complete variant dataset using only short-read data. However, the conclusions of this study may be limited to human data because GATK requires known SNV/indel sites for VQSR/BQSR, and deep learning-based algorithms such as DeepVariant, PEPPER, and NanoCaller require custom models trained for nonhuman species.

## Supplementary information

Supplementary Figures&Tables

Supplementary Note
