# Supplementary material for: Comparative evaluation of SNVs, indels, and structural variations detected with short- and long-read sequencing data
Source: Hum Genome Var. 2024 Apr 17;11:18. doi: 10.1038/s41439-024-00276-x (PMC11024196; doi:10.1038/s41439-024-00276-x)
Supplement: Supplementary file 1 — Supplementary Figures&Tables [file 41439_2024_276_MOESM1_ESM.pdf]

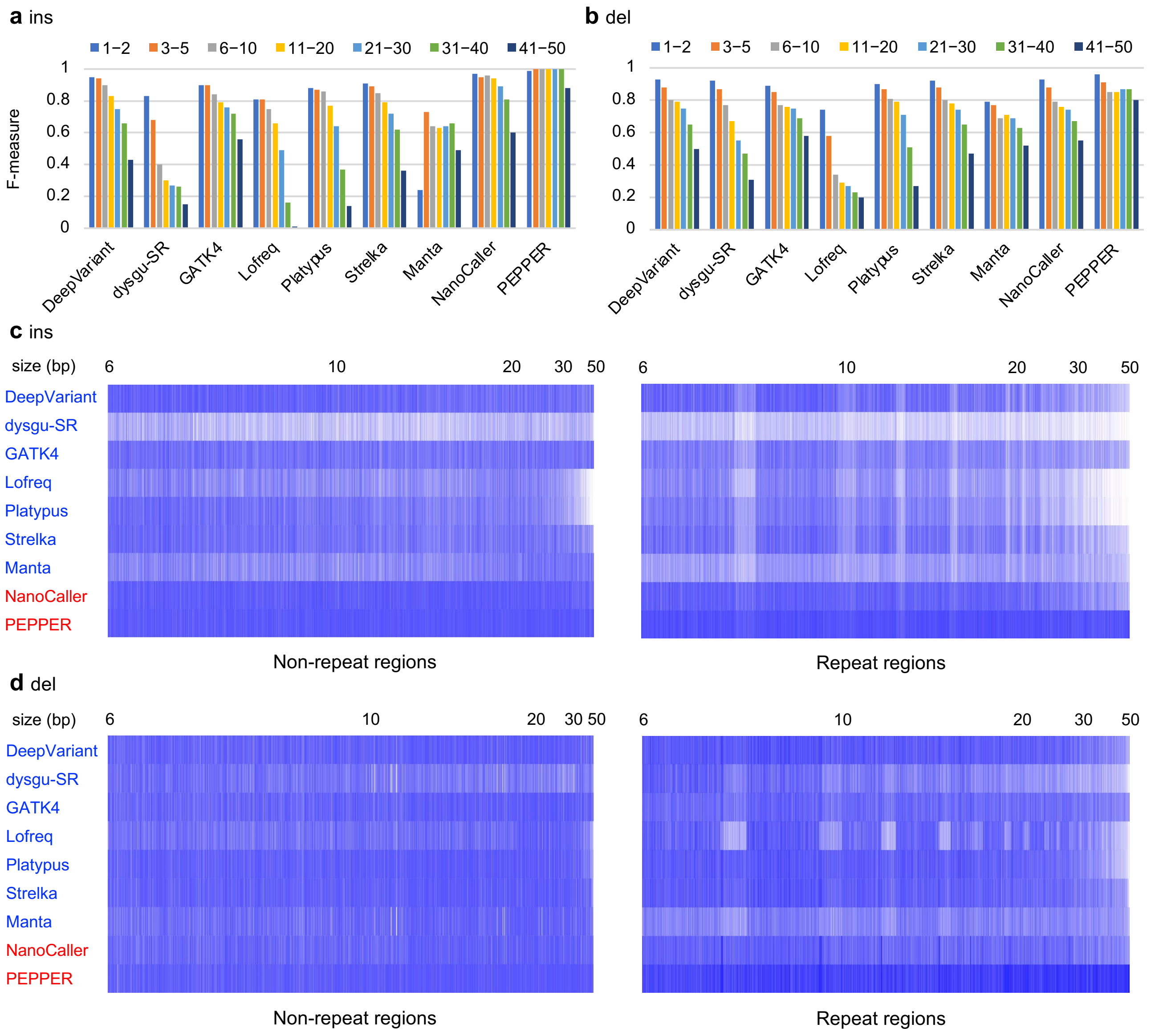

Figure S1. Evaluation of insertion calls by size in HG002.
(**a**) Accuracy by size for indel insertion calls in HG002. F-measures of ins calls for the indicated short read-based and long read-based algorithms are shown in color bars over the ins-size ranges (blue: 1 and 2 bp, orange: 3−5 bp, grey: 6−10 bp, yellow: 11−20 bp, cyan: 21−30 bp, green: 31−40 bp, black: 41−50 bp). (**b**) Accuracy by size for indel deletion calls in HG002. F-measures of del calls for the indicated algorithms are shown as in (a). (**c**) Size-based distribution of 6−50 bp ins TP calls matched with the HG002 reference. Of HG002 inss calls from the indicated short read-based (shown in blue) and long read-based (shown in red) indel detection algorithms, TP calls that matched with the reference inss are plotted as blue bars. The x-axis shows the reference inss sorted by size, with representative sizes shown at the top. White blanks indicate the reference inss that were not detected by the corresponding algorithm. The left and the right panels show the inss in non-repetitive and repetitive regions, respectively. To focus on large indels, only indels in 6−50 bp range are shown. (**d**) Size-based distribution of 6−50 bp del TP calls matched with the HG002 reference across the size. The plots are shown as in (c).

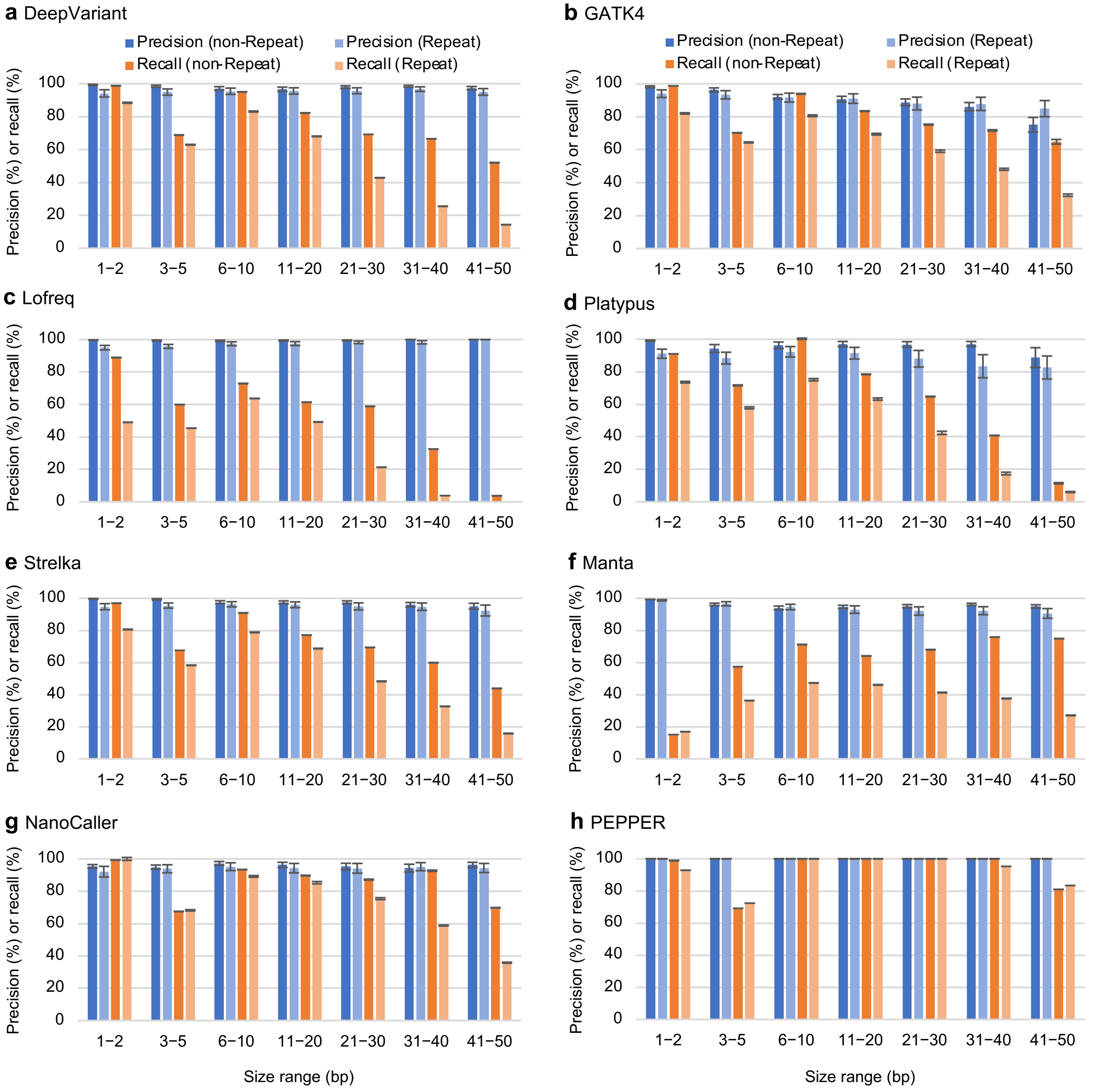

Figure S2. Precision and recall of insertion calls by size in NA12878.
(**a-f**) Insertions (inss) of NA12878 were evaluated for short read-based indel detection algorithms, DeepVariant (**a**), GATK4 (**b**), Lofreq (**c**), Platypus (**d**), Strelka (**e**), and Manta (**f**) using Illumina WGS data. Ins calls in non-repetitive and repetitive regions were divided into the indicated size ranges to determine precision and recall. Blue and light blue bars indicate precision values of SNVs present in non-repetitive and repetitive regions, respectively. Orange and light orange bars indicate recall values of SNVs present in non-repetitive and repetitive regions, respectively. The confident interval with each bar is based on the estimated errors from manual visual inspection of 50 variants. (**g,h**) Ins calls of NA12878 were evaluated for long read-based indel detection algorithms, NanoCaller (**g**) and PEPPER/DeepVariant (**h**) using PacBio HiFi WGS data.

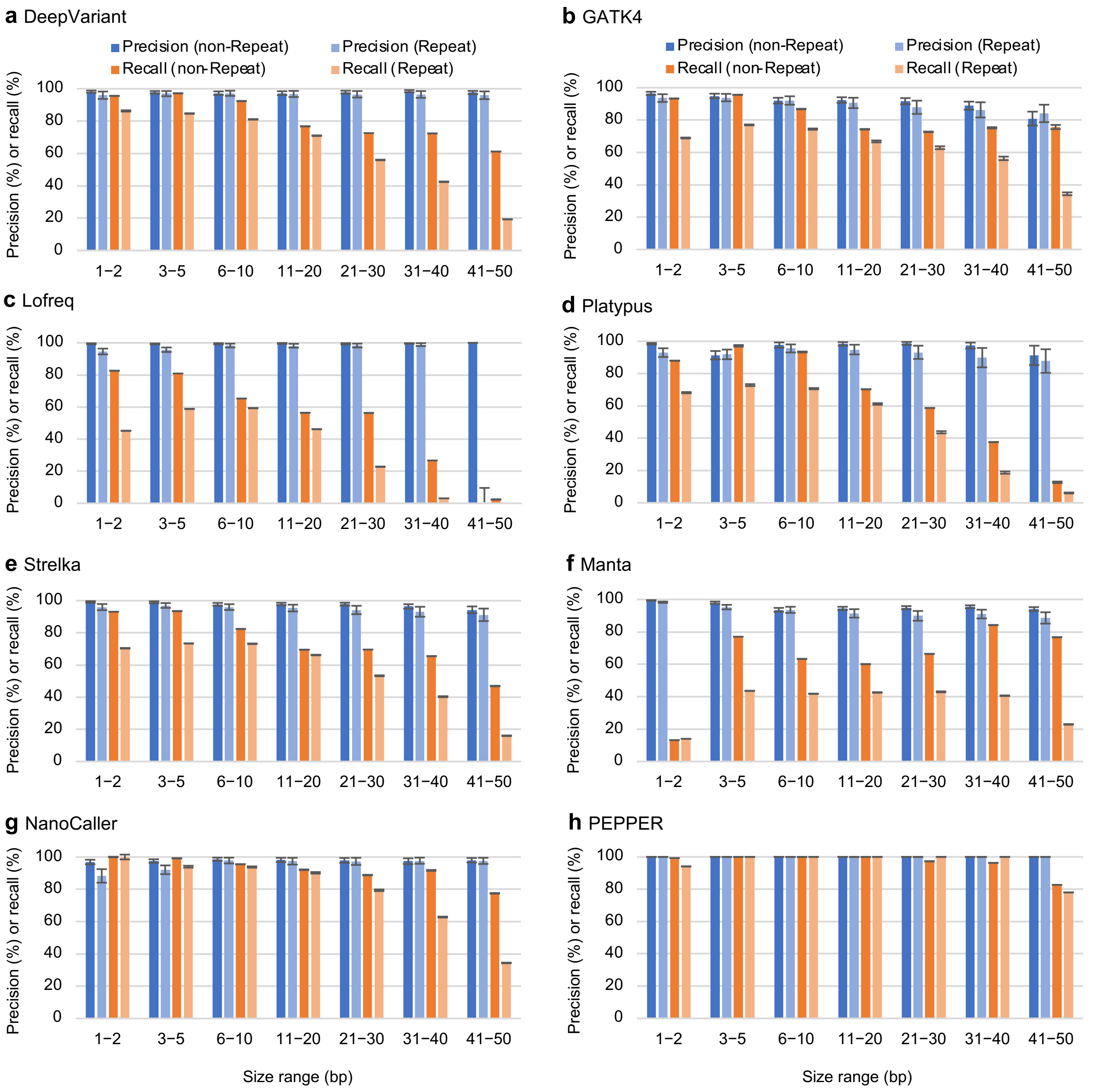

Figure S3. Precision and recall of insertion calls by size in HG002.
(**a-f**) Insertions (inss) of HG002 were evaluated for short read-based indel detection algorithms, DeepVariant (**a**), GATK4 (**b**), Lofreq (**c**), Platypus (**d**), Strelka (**e**), and Manta (**f**) using Illumina WGS data. Ins calls in non-repetitive and repetitive regions were divided into the indicated size ranges to determine precision and recall. Bars are shown as in Fig. S2. (**g,h**) Ins calls of HG002 were evaluated for long read-based indel detection algorithms, NanoCaller (**g**) and PEPPER/DeepVariant (**h**) using PacBio HiFi WGS data.

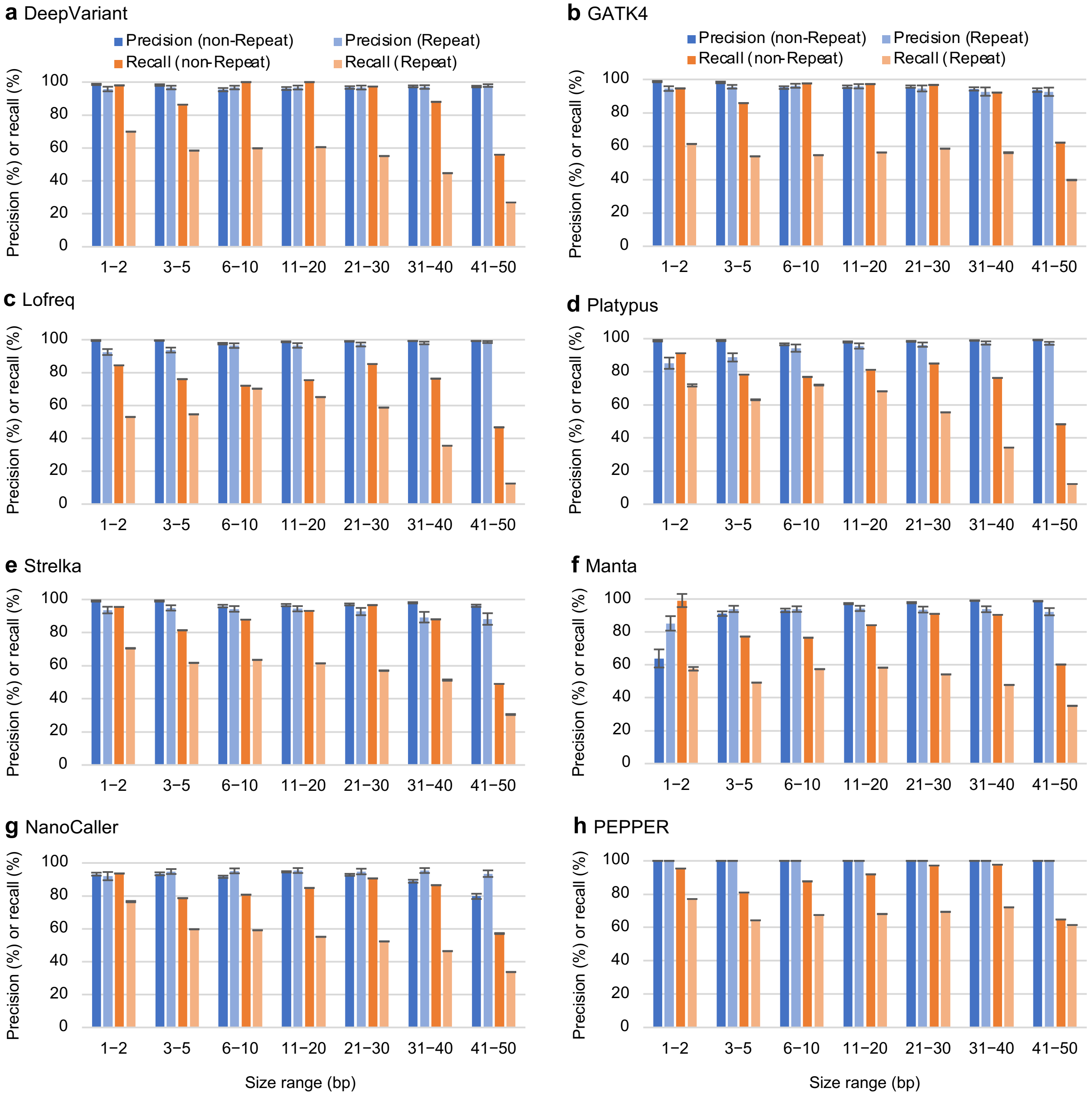

Figure S4. Precision and recall of deletion calls by size in NA12878.
(**a-f**) Deletions (dels) of NA12878 were evaluated for short read-based indel detection algorithms, DeepVariant (**a**), GATK4 (**b**), Lofreq (**c**), Platypus (**d**), Strelka (**e**), and Manta (**f**) using Illumina WGS data. Del calls in non-repetitive and repetitive regions were divided into the indicated size ranges to determine precision and recall. Bars are shown as in Fig. S2. (**g,h**) Del calls of NA12878 were evaluated for long read-based indel detection algorithms, NanoCaller (**g**) and PEPPER/DeepVariant (**h**) using PacBio HiFi WGS data.

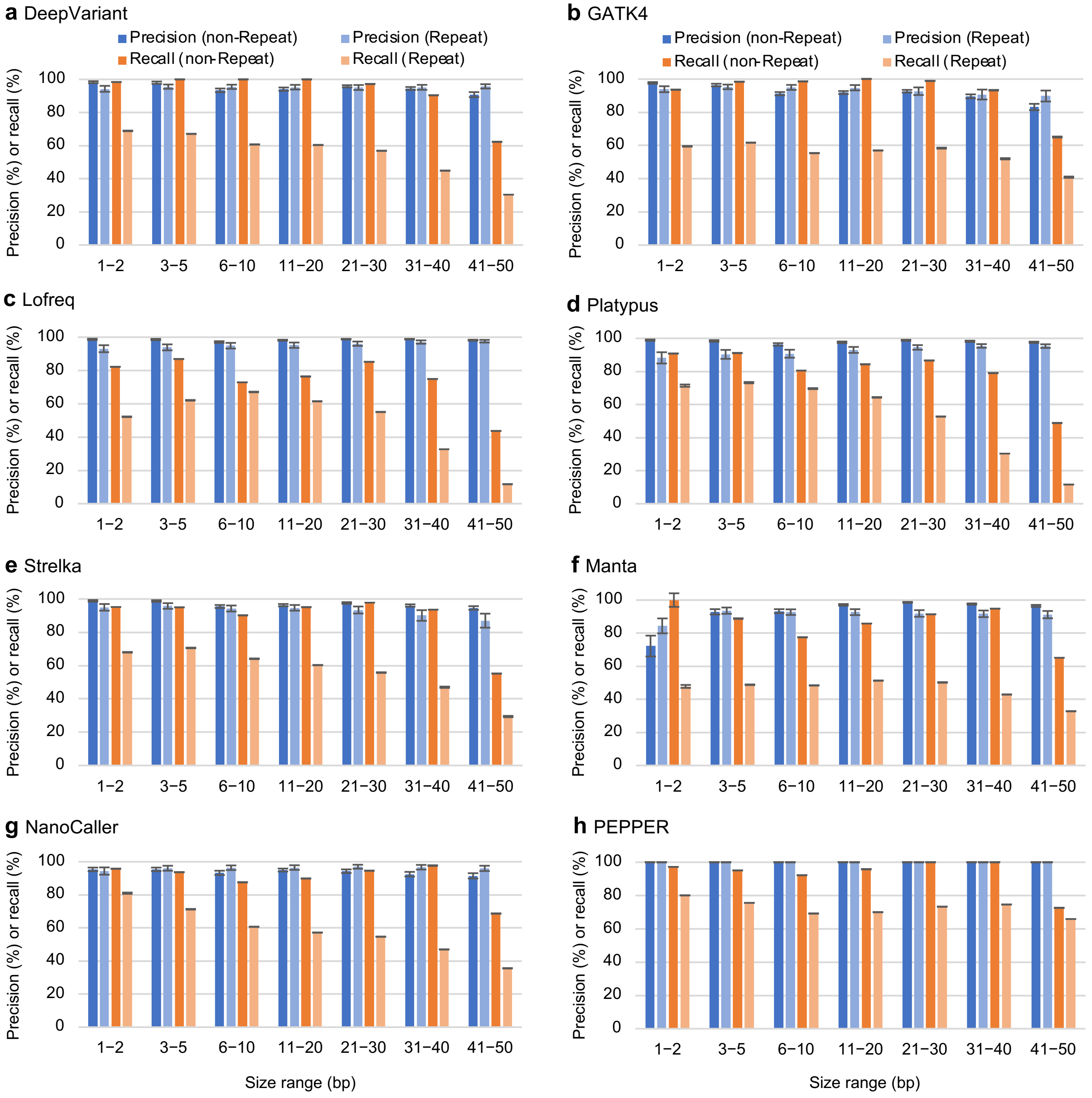

Figure S5. Precision and recall of deletion calls by size in HG002.
(**a-f**) Deletions (dels) of HG002 were evaluated for short read-based indel detection algorithms, DeepVariant (**a**), GATK4 (**b**), Lofreq (**c**), Platypus (**d**), Strelka (**e**), and Manta (**f**) using Illumina WGS data. Del calls in non-repetitive and repetitive regions were divided into the indicated size ranges to determine precision and recall. Bars are shown as in Fig. S2. (**g,h**) Del calls of HG002 were evaluated for long read-based indel detection algorithms, NanoCaller (**g**) and PEPPER/DeepVariant (**h**) using PacBio HiFi WGS data.

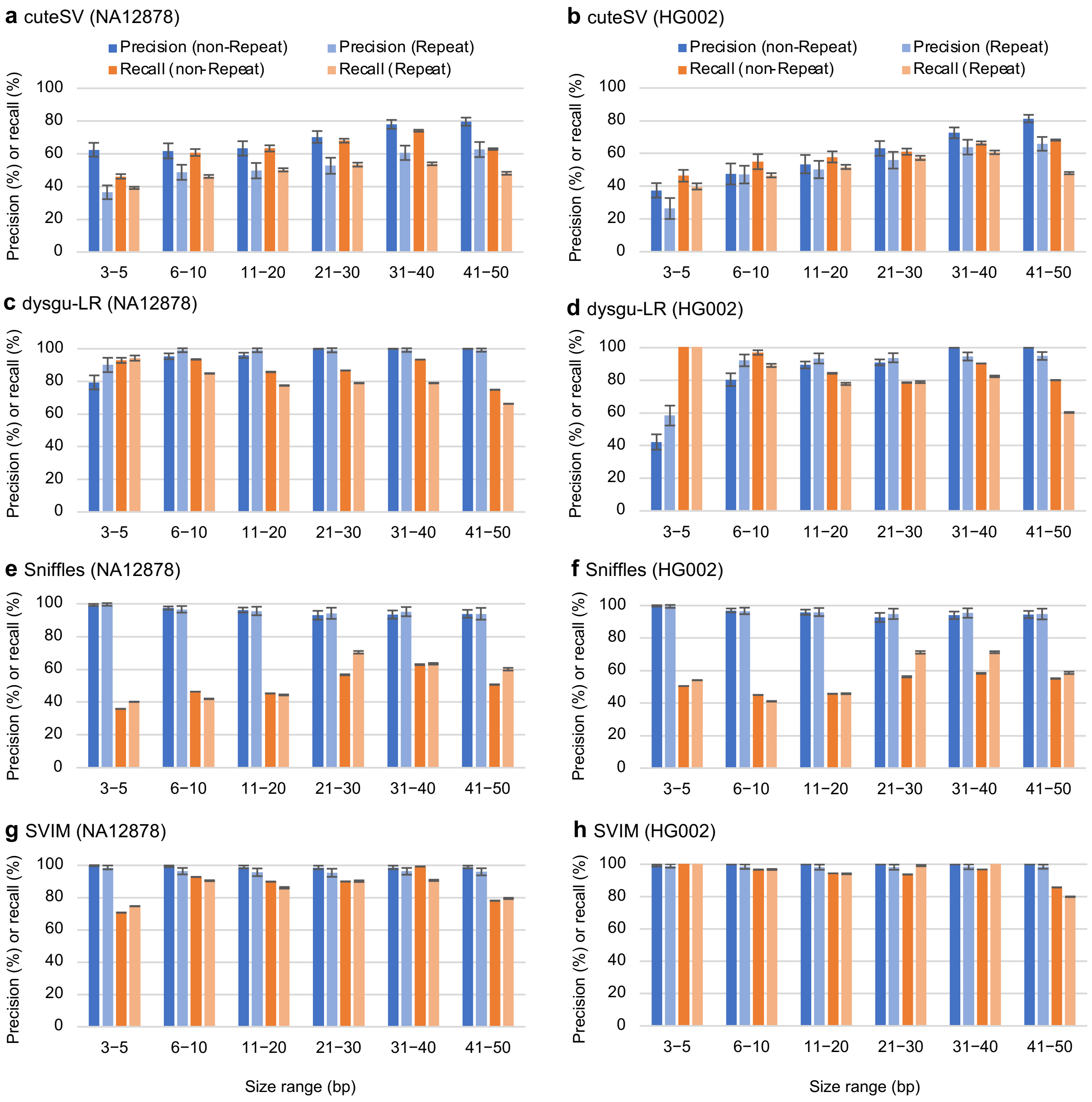

Figure S6. Precision and recall of insertion calls by size for long read-based indel/SV detection algorithms.
Insertions (inss) were evaluated for long read-based indel/SV detection algorithms, cuteSV (**a,b**), Dysgu (**c,d**), Sniffles (**e.f**), and SVIM (**g,h**) using NA12878 (**a,c,e,g**) and HG002 (**b,d,f,h**) PacBio HiFi long read WGS data. Ins calls in non-repetitive and repetitive regions were divided into the indicated size ranges to determine precision and recall. Bars are shown as in Fig. S2.

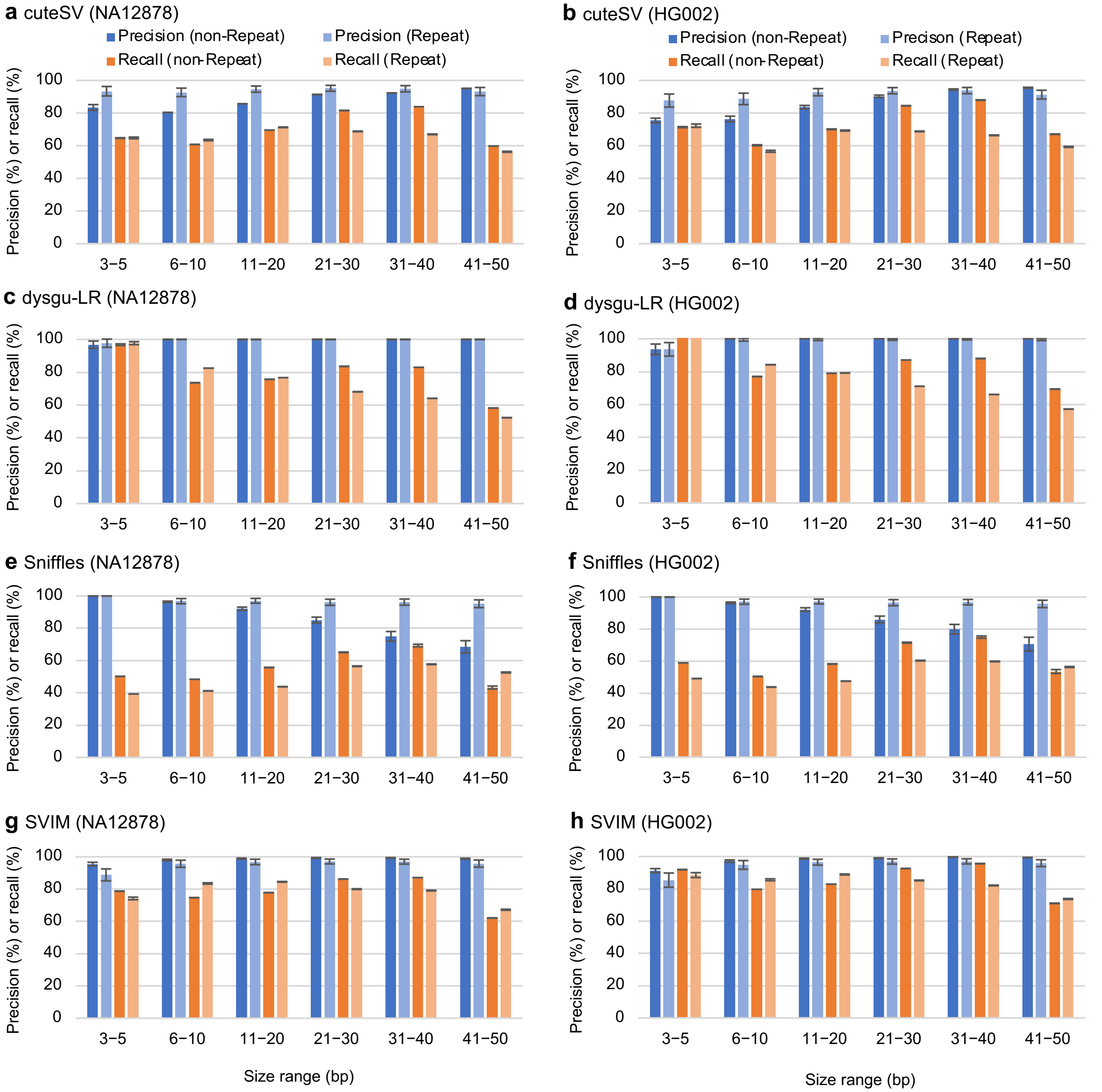

Figure S7. Precision and recall of deletion calls by size for long read-based indel/SV detection algorithms.
Deletions (dels) were evaluated for long read-based indel/SV detection algorithms, cuteSV (**a**,**b**), Dysgu (**c**,**d**), Sniffles (**e.f**), and SVIM (**g**,**h**) using NA12878 (**a**,**c**,**e**,**g**) and HG002 (**b**,**d**,**f**,**h**) PacBio HiFi long read WGS data. Del calls in non-repetitive and repetitive regions were divided into the indicated size ranges to determine precision and recall. Bars are shown as in Fig. S2.

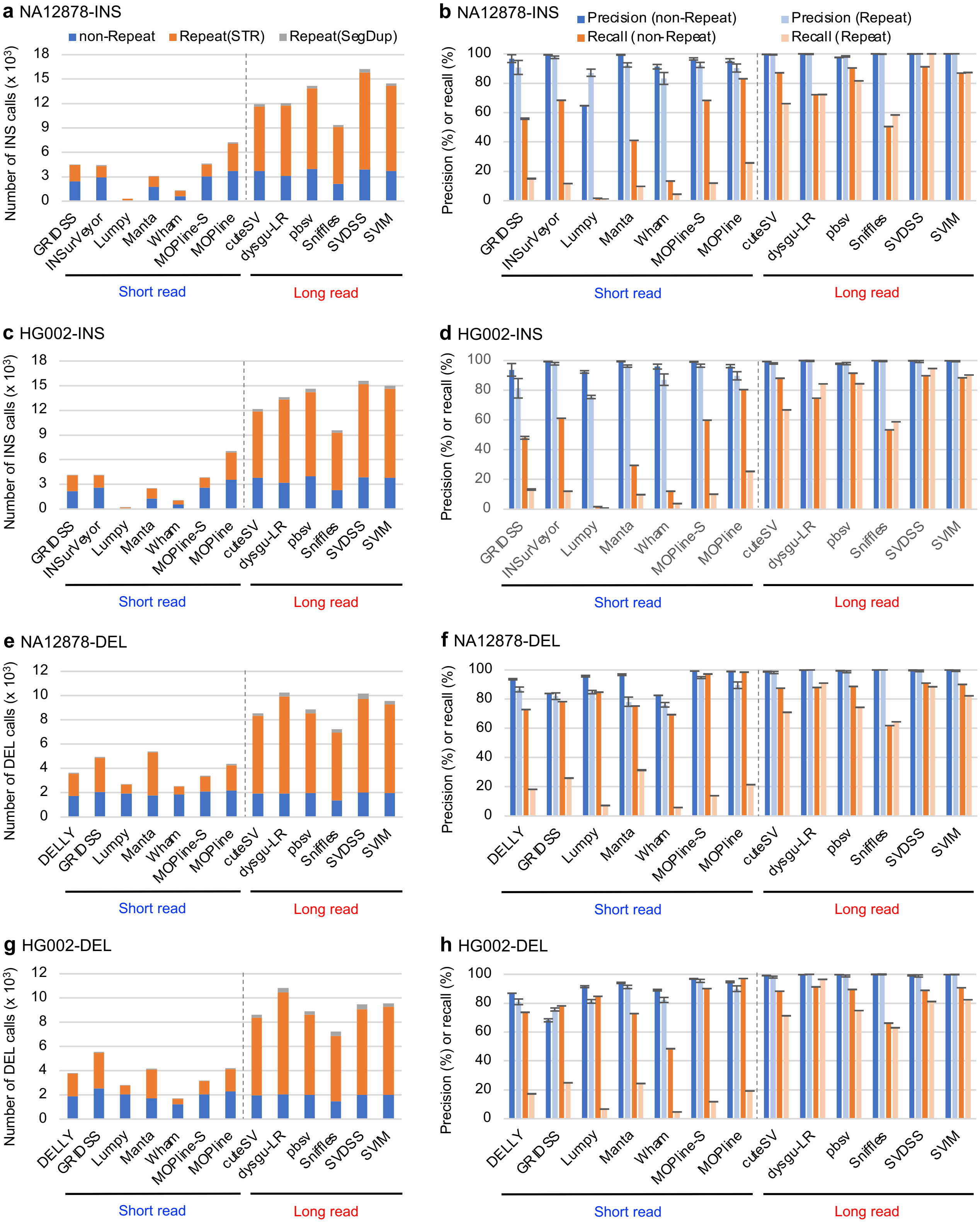

Figure S8. Evaluation of SVs called with short read-based and long read-based SV detection algorithms in NA12878 and HG002.

(**a**) Number of INS calls for NA12878. INSs and DUPs of ≧ 50 bp were detected with the indicated short read-based (GRIDSS, INSurVeyor, Lumpy, Manta, Wham, MOPline-S, and MOPline) and long read-based (cuteSV, dysugu-LR, pbsv, Sniffles, SVDSS, and SVIM) SV detection algorithms using NA12878 short read or long read WGS data. DUP calls were converted to INSs. Bars are shown as in Fig. 1. MOPline-S indicates MOPline-7t in the single sample mode. (**b**) Precision and recall of INS calls for NA12878. Bars are shown as in Fig. 2. (**c**) Number of INS calls for HG002. (**d**) Precision and recall of INS calls for HG002. (**e**) Number of DEL calls of ≧ 50 bp for NA12878. (**f**) Precision and recall of DEL calls for NA12878. (**g**) Number of DEL calls for H002. (**h**) Precision and recall of DEL calls for HG002.

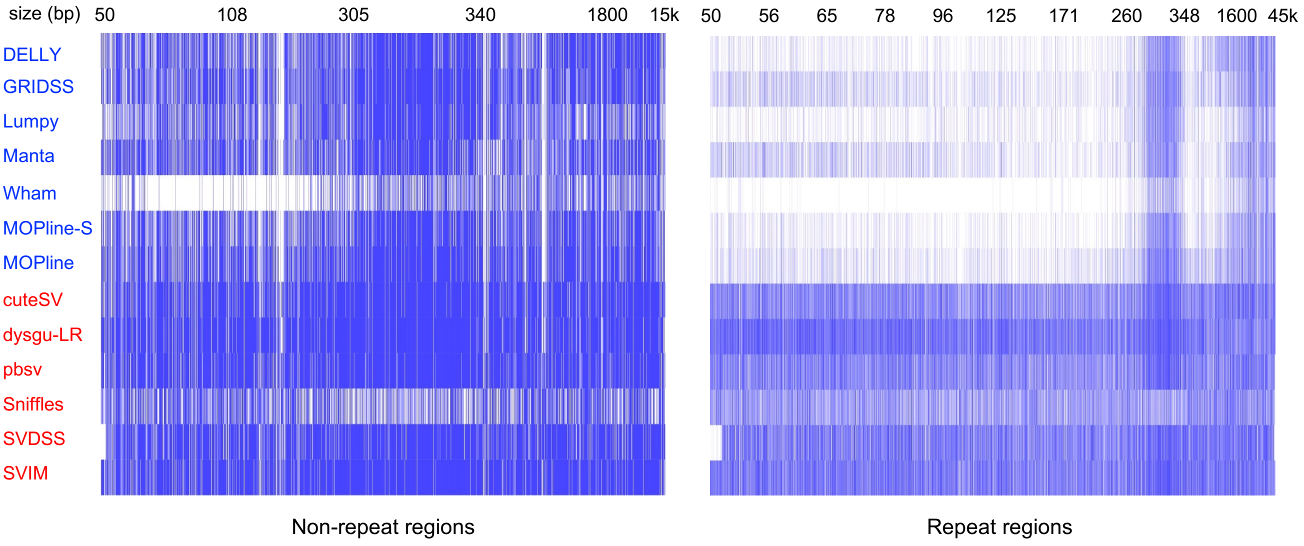

Figure S9. Distribution of DEL TP calls matched with the HG002 reference across the size. Of HG002 DEL calls from the indicated short read-based (marked with blue letters) and long read-based (marked with red letters) SV detection algorithms, the reference DELs (i.e., TP calls) that matched with the DEL calls were plotted as blue bars. The x-axis shows the reference DELs sorted by size, with representative sizes shown at the top. White blanks indicate the reference DELs that were not detected by the corresponding algorithm. The left and the right panels show the DELs in non-repetitive and repetitive regions, respectively.

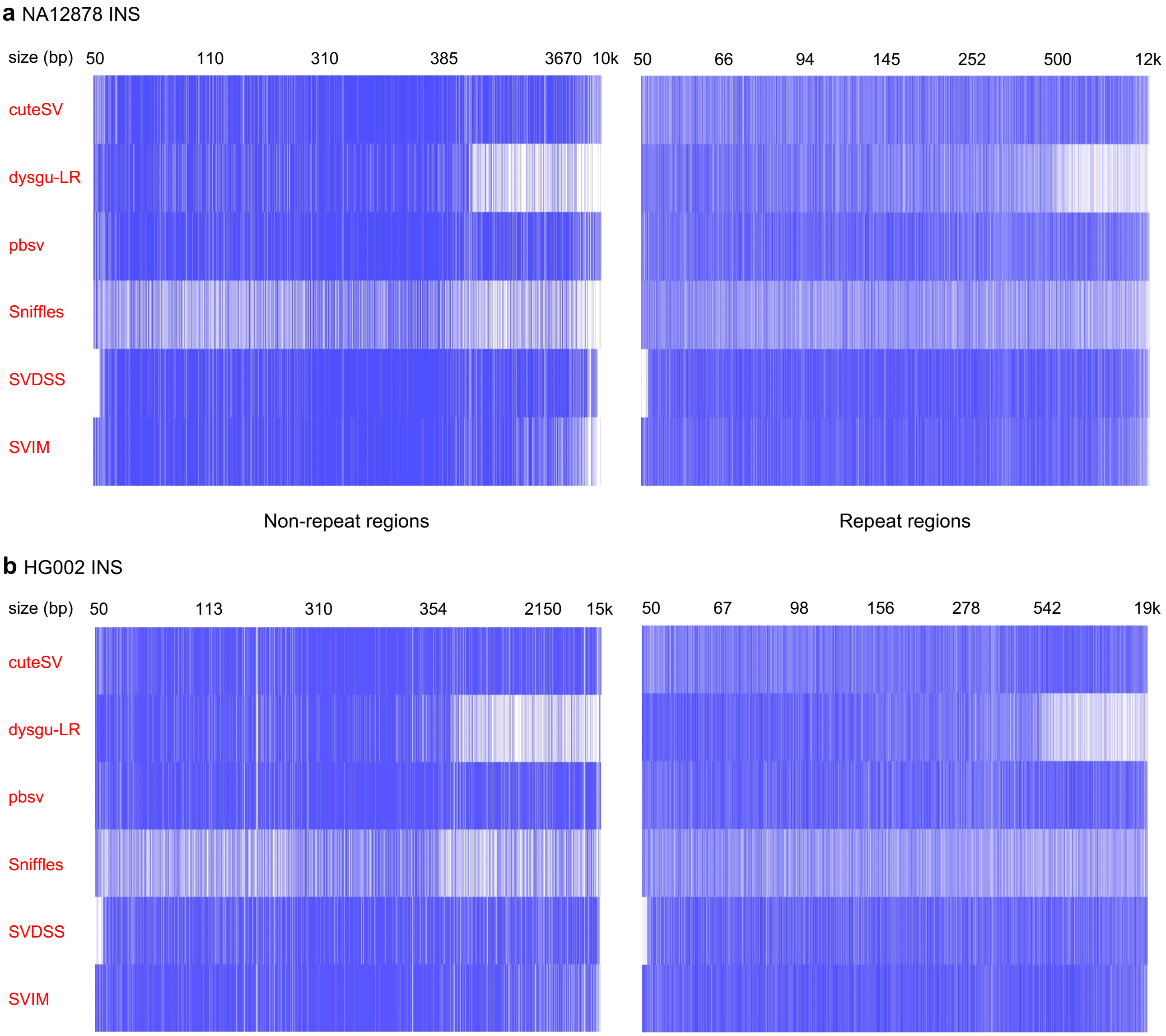

Figure S10. Distribution of INS TP calls matched with the NA12878 and HG002 references across the size.
Of NA12878 (**a**) or HG002 (**b**) INSs calls from the indicated long read-based SV detection algorithms, the reference INSs (i.e., TP calls) that matched with the INS calls were plotted as blue bars. The x-axis shows the reference INSs sorted by size, with representative sizes shown at the top. White blanks indicate the reference INSs that were not detected by the corresponding algorithm. The left and the right panels show the INSs in non-repetitive and repetitive regions, respectively.

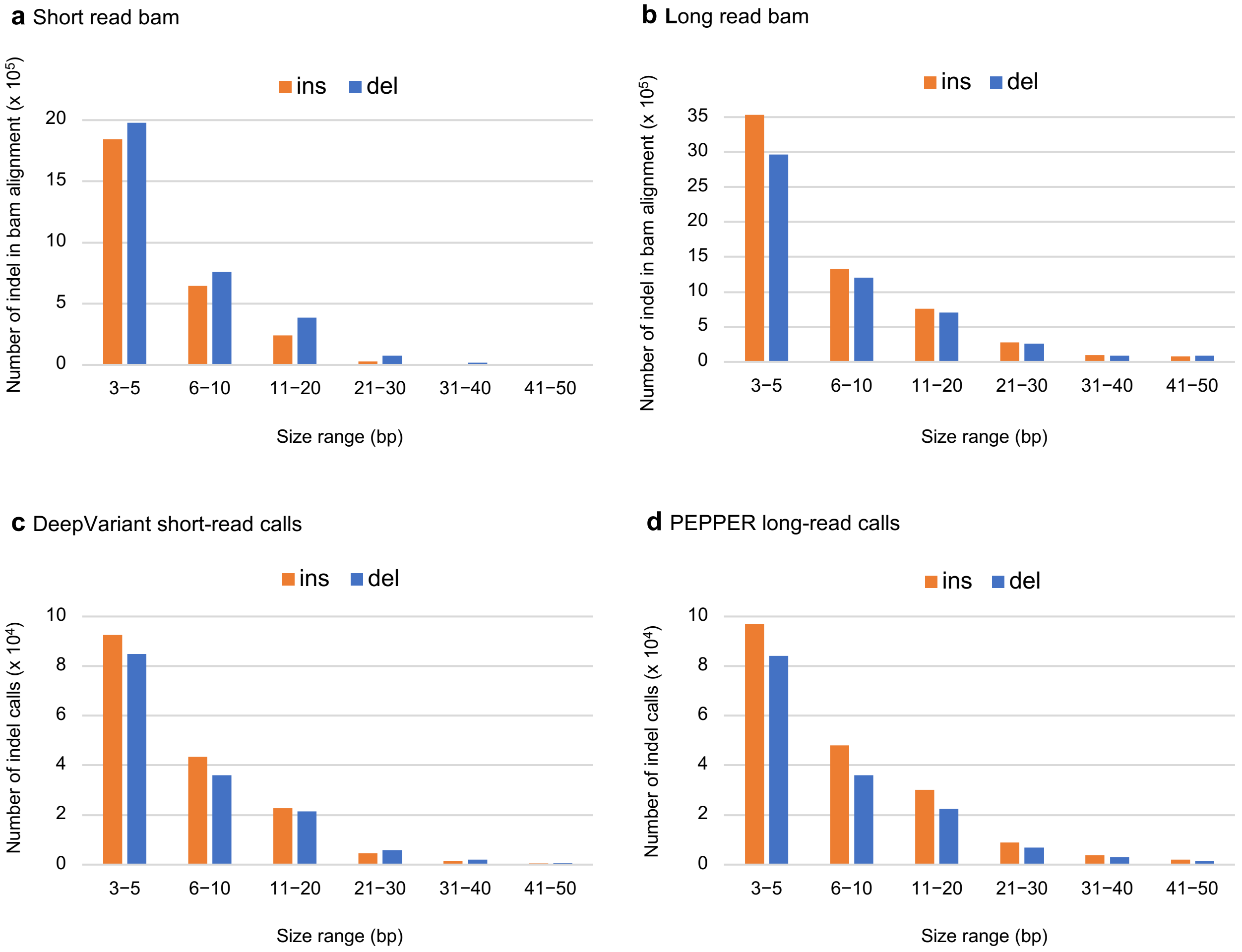

Figure S11. Indel size-frequency in short-read and long-read alignments.
(**a**) Number of indels observed in short read bam alignments. A bam file was generated with NA12878 WGS data of 150 bp paired-end reads using bwa mem. Ins and del alignments in the CIGAR strings of the short read bam were counted and their frequencies are shown across the indicated size ranges of indel. Orange and blue bars indicate the number of ins and del, respectively. (**b**) Number of indels observed in long read bam alignments. A bam file was generated with NA12878 WGS data of PacBio HiFi long reads using Minimap2. Ins and del alignments in the CIGAR strings of the bam were counted and shown as in (a). (**c**) Number of indel calls of DeepVariant. Indel calls of short read-based algorithm, DeepVariant, were obtained with the short read bam used in (a). (**d**) Number of indel calls of PEPPER. Indel calls of the log read-based algorithm, PEPPER, were obtained with the long read bam used in (b).

Supplementary Table S1 WGS datasets

Sample	Read length	Genome coverage	Platform	Accession number
NA12878	150 bp × 2	36.7×	Illumina NovaSeq 6000	ERR3239334 (by 1000 Genomes Project)
HG002	148 bp × 2	30.0×	Illumina HiSeq 2500	SRR1766465~ SRR1766595 (by GIAB)
NA12878 (PacBio HiFi)	10.0 Kb	29.2×	PacBio Sequel II	SRR9001768-SRR9001773 (by PacBio)
HG002 (PacBio HiFi)	19.1 Kb	39.9×	PacBio Sequel II	SRR18355743, SRR18355744, SRR18358816, SRR18358817 (by UCSC Genomics Institute)

Supplementary Table S2 Reference variant sets of NA12878 and HG002

Sample	Genomic region	SNV	Indel		SV	
			ins	del	INS	DEL
NA12878	Total	3,838,837	506,003	524,767	16,569	11,336
	Non-repeat	3,430,135	281,463	282,628	4,261	2,210
	Repeat (STR)	203,042	206,467	223,504	11,549	8,455
	Repeat (SegDup)	205,660	18,073	18,635	759	671
HG002	Total	3,793,041	495,779	512,330	16,915	11,796
	Non-repeat	3,432,201	277,097	278,032	4,473	2,219
	Repeat (STR)	187,305	201,226	216,598	11,792	9,027
	Repeat (SegDup)	173,535	18,266	17,700	650	550

Supplementary Table S3 Numerical data of evaluation results for SNV calling

Sample	Read type	Algorithm	Number of calls				TP1 *1		Precision1 (%) *2		Recall1 (%) *3		FP *4		TPR in FP (%) *5		TP2 *6		Precision2 (%) *7		Recall2 (%) *8		F-measure *9	
			Total	Non-repeat	Repeat		Non-repeat	Repeat	Non-repeat	Repeat	Non-repeat	Repeat	Non-repeat	Repeat	Non-repeat	Repeat	Non-repeat	Repeat	Non-repeat	Repeat	Non-repeat	Repeat	Non-repeat	Repeat
					STR	SegDup																		
NA12878	Short	DeepVariant	3794364	3419529	172303	202532	3394591	288546	99.3	77	99	70.6	24938	86289	88	69 (STR), 81 (SegDup)	3416536	354832	99.9	94.7	98.2	59.6	0.99	0.73
		GATK4	3823431	3424809	155726	242896	3386496	278692	98.9	69.9	98.7	68.2	38313	119930	50	67 (STR), 73 (SegDup)	3405653	364631	99.4	91.5	97.9	61.3	0.99	0.73
		Lofreq	3764908	3395796	161373	207739	3360223	261381	99	70.8	98	64	35573	107731	54	48 (STR), 73 (SegDup)	3379432	329715	99.5	89.3	97.1	55.4	0.98	0.68
		Strelka	3978371	3458983	262875	256513	3397953	305484	98.2	58.8	99.1	74.7	61030	213904	56	38 (STR), 79 (SegDup)	3432130	430011	99.2	82.8	98.6	72.3	0.99	0.77
	Long	NanoCaller	4103899	3480030	304898	318971	3414844	342293	98.1	54.9	99.6	83.8	65186	281576	100	97 (STR), 84 (SegDup)	3480030	594908	100	95.4	100	100	1	0.98
		PEPPER	3999298	3462778	254757	281763	3418063	332782	98.7	62	99.6	81.4	44715	203738	94	95 (STR), 85 (SegDup)	3460095	514187	99.9	95.8	99.4	86.4	1	0.91
HG002	Short	DeepVariant	3823525	3443788	174810	204927	3406540	263246	98.9	69.3	99.3	73	37248	116491	88	87 (STR), 90 (SegDup)	3439318	366913	99.9	96.6	98	58.9	0.99	0.73
		GATK4	3827157	3440981	155468	230708	3392063	246799	98.6	63.9	98.8	68.4	48918	139377	64	58 (STR), 76 (SegDup)	3423371	346289	99.5	89.7	97.5	55.6	0.98	0.69
		Lofreq	3723740	3383010	151067	189663	3339330	225785	98.7	66.3	97.3	62.6	43680	114945	56	64 (STR), 69 (SegDup)	3363791	302952	99.4	88.9	95.8	48.7	0.98	0.63
		Strelka	4193509	3634129	284033	275347	3407429	276365	93.8	49.4	99.3	76.6	226700	283015	20	38 (STR), 65 (SegDup)	3452769	423189	95	75.7	98.4	68	0.97	0.72
	Long	NanoCaller	4145977	3510443	304878	330656	3429548	319677	97.7	50.3	99.9	88.6	80895	315857	100	100 (STR), 93 (SegDup)	3510443	622567	100	98	100	100	1	0.99
		PEPPER	4019922	3481966	254945	283011	3427512	310342	98.4	57.7	99.9	86	54454	227614	96	100 (STR), 85 (SegDup)	3479788	517194	99.9	96.1	99.1	83.1	0.99	0.89

*1 True positive (TP) calls that matched with the reference variants.

*2 Precision calculated with TP1 and the number of calls.

*3 Recall calculated with TP1 and the number of the reference variant.

*4 The false positive (FP) calls calculated by substitution of TP1 from the number of calls.

*5 True positive rate estimated in false positive calls by manual visual inspection of 100~200 variants randmly selected from FP calls.

*6 Corrected TP calls, which are sum of TP1 and TP estimates contained in FPs.

*7 Corrected precision calculated with TP2 and the number of calls.

*8 Corrected recall calculated with TP2 and the number of the reference variant.

*9 F-measure = 2 x Precision2 x Recall2 * 0.01 / (Precision2 + Recall2).

Supplementary Table S4 Numerical data of evaluation results for for indel calling

Sample	Read type	Algorithm	Variant type	Number of calls				TP1 *1		Precision1 (%) *2		Recall1 (%) *3		FP *4		TPR in FP (%) *5		TP2 *6		Precision2 (%) *7		Recall2 (%) *8		F-measure *9	
				Total	Non-repeat	Repeat		Non-repeat	Repeat	Non-repeat	Repeat	Non-repeat	Repeat	Non-repeat	Repeat	Non-repeat	Repeat	Non-repeat	Repeat	Non-repeat	Repeat	Non-repeat	Repeat	Non-repeat	Repeat
						STR	SegDup																		
NA12878	Short	DeepVariant	ins	491776	280510	197641	13625	270930	179092	96.8	84.8	96.3	79.8	9580	32174	70 (1-5 bp), 64 (> 5 bp)	64 (1-5 bp), 70 (> 5 bp)	277026	200574	98.8	94.9	91.5	76.3	0.95	0.85
			del	448373	284256	150104	14013	274462	147811	96.6	90.1	97.1	61	9794	16306	54 (1-5 bp), 40 (> 5 bp)	62 (1-5 bp), 64 (> 5 bp)	279561	158006	98.3	96.3	96.1	63	0.97	0.76
		dysgu-SR	ins	373015	242878	118861	11276	231998	111071	95.5	85.3	82.4	49.5	10880	19066	62 (1-5 bp), 70 (> 5 bp)	68 (1-5 bp), 58 (> 5 bp)	238789	123860	98.3	95.2	78.9	47.1	0.88	0.63
			del	464117	264354	186314	13449	86750	115345	32.8	57.7	30.7	47.6	177604	84418	90 (1-5 bp), 34 (> 5 bp)	90 (1-5 bp), 74 (> 5 bp)	246362	182448	93.2	91.3	84.7	72.7	0.89	0.81
		GATK4	ins	496647	286376	194973	15298	269199	169932	94	80.8	95.6	75.7	17177	40339	58 (1-5 bp), 30 (> 5 bp)	66 (1-5 bp), 58 (> 5 bp)	278233	195636	97.2	93	91.9	74.5	0.94	0.83
			del	426850	275616	135448	15786	267321	134544	97	89	94.6	55.6	8295	16690	50 (1-5 bp), 28 (> 5 bp)	54 (1-5 bp), 60 (> 5 bp)	271201	143852	98.4	95.1	93.2	57.3	0.96	0.72
		Lofreq	ins	378033	246056	120544	11433	241460	119458	98.3	90.5	85.8	53.2	4596	12519	80 (1-5 bp), 78 (> 5 bp)	52 (1-5 bp), 74 (> 5 bp)	244764	126940	99.5	96.2	80.9	48.3	0.89	0.64
			del	392172	240718	139210	12244	235275	133493	97.7	88.1	83.2	55.1	5443	17961	78 (1-5 bp), 35 (> 5 bp)	44 (1-5 bp), 68 (> 5 bp)	239345	142741	99.4	94.2	82.3	56.9	0.9	0.71
		Platypus	ins	460521	267529	177388	15604	248770	147225	93	76.3	88.4	65.6	18759	45767	70 (1-5 bp), 78 (> 5 bp)	56 (1-5 bp), 70 (> 5 bp)	262204	174683	98	90.5	86.6	66.5	0.92	0.77
			del	450217	259852	173669	16696	250831	151621	96.5	79.6	88.7	62.6	9021	38744	66 (1-5 bp), 29 (> 5 bp)	40 (1-5 bp), 66 (> 5 bp)	256568	169191	98.7	88.9	88.2	67.4	0.93	0.77
		Strelka	ins	469290	273678	181521	14091	265785	168855	97.4	86.3	94.4	75.2	7893	26757	86 (1-5 bp), 62 (> 5 bp)	64 (1-5 bp), 72 (> 5 bp)	271617	186970	99.2	95.6	89.7	71.2	0.94	0.82
			del	446116	272700	158333	15083	264068	149375	96.8	86.1	93.4	61.7	8632	24041	70 (1-5 bp), 33 (> 5 bp)	58 (1-5 bp), 54 (> 5 bp)	269762	163048	98.9	94	92.7	65	0.96	0.77
		Manta	ins	170733	89214	77215	4304	84705	72675	94.9	89.2	30.1	32.4	4509	8844	36 (1-5 bp), 33 (> 5 bp)	68 (1-5 bp), 58 (> 5 bp)	86134	78035	96.5	95.7	28.5	29.7	0.44	0.45
			del	556414	403392	133380	19642	235273	115822	58.3	75.7	83.2	47.8	168119	37200	23 (1-5 bp), 24 (> 5 bp)	60 (1-5 bp), 52 (> 5 bp)	273936	137652	67.9	90	94.2	54.9	0.79	0.68
	Long	NanoCaller	ins	536883	293020	221630	22233	267016	185191	91.4	75.9	94.9	82.5	26004	58672	44 (1-5 bp), 70 (> 5 bp)	70 (1-5 bp), 78 (> 5 bp)	278844	227941	95.2	93.5	92.1	86.7	0.94	0.9
			del	457316	282047	153570	21699	258122	145844	91.5	83.2	91.3	60.2	23925	29425	20 (1-5 bp), 8 (> 5 bp)	62 (1-5 bp), 60 (> 5 bp)	262744	163977	93.2	93.6	90.3	65.4	0.92	0.77
		PEPPER	ins	514291	281226	215188	17877	273501	203417	97.3	87.3	97.2	90.6	7725	29648	100 (1-5 bp), 100 (> 5 bp)	100 (1-5 bp), 100 (> 5 bp)	281226	233065	100	100	92.9	88.7	0.96	0.94
			del	446669	269278	160288	17103	265066	171910	98.4	96.9	93.8	71	4212	5481	100 (1-5 bp), 100 (> 5 bp)	100 (1-5 bp), 100 (> 5 bp)	269278	177391	100	100	92.6	70.7	0.96	0.83
HG002	Short	DeepVariant	ins	484265	280049	190792	13424	264165	167991	94.6	82.3	95.3	76.5	264165	167991	64 (1-5 bp), 70 (> 5 bp)	80 (1-5 bp), 82 (> 5 bp)	273969	197737	97.8	96.8	94.8	82	0.96	0.89
			del	442578	282014	146737	13827	268996	141530	95.4	88.1	96.8	60.4	268996	141530	60 (1-5 bp), 26 (> 5 bp)	58 (1-5 bp), 56 (> 5 bp)	276273	152471	98	95	98.6	65.1	0.98	0.77
		dysgu-SR	ins	324991	224133	91109	9749	215627	87454	96.2	86.7	77.8	39.8	8506	13404	80 (1-5 bp), 66 (> 5 bp)	60 (1-5 bp), 78 (> 5 bp)	222381	95715	99.2	94.9	76.9	39.7	0.87	0.56
			del	441672	267939	161316	12417	84563	100908	31.6	58.1	30.4	43.1	183376	72825	96 (1-5 bp), 60 (> 5 bp)	85 (1-5 bp), 92 (> 5 bp)	260464	160065	97.2	92.1	93	68.3	0.95	0.78
		GATK4	ins	462478	279003	169265	14210	256500	147272	91.9	80.3	92.6	67.1	22503	36203	50 (1-5 bp), 40 (> 5 bp)	66 (1-5 bp), 60 (> 5 bp)	267430	170485	95.9	92.9	92.5	70.7	0.94	0.8
			del	418947	272931	130948	15068	260139	125784	95.3	86.1	93.6	53.7	12792	20232	42 (1-5 bp), 14 (> 5 bp)	60 (1-5 bp), 58 (> 5 bp)	264982	137802	97.1	94.4	94.6	58.8	0.96	0.72
		Lofreq	ins	356096	233624	112386	10086	226342	108145	97.1	88.3	81.7	49.3	7282	14327	78 (1-5 bp), 84 (> 5 bp)	58 (1-5 bp), 86 (> 5 bp)	231710	117798	99.2	96.2	80.2	48.8	0.89	0.65
			del	377247	234065	132032	11150	227530	123826	97.2	86.5	81.8	52.8	6535	19356	52 (1-5 bp), 26 (> 5 bp)	54 (1-5 bp), 60 (> 5 bp)	230813	134631	98.6	94	82.4	57.5	0.9	0.71
		Platypus	ins	437337	263366	159350	14621	245102	133910	93.1	77	88.5	61	18264	40061	54 (1-5 bp), 86 (> 5 bp)	66 (1-5 bp), 82 (> 5 bp)	255977	162182	97.2	93.2	88.5	67.2	0.93	0.78
			del	436022	256151	163978	15893	248432	142437	97	79.2	89.4	60.8	7719	37434	58 (1-5 bp), 35 (> 5 bp)	52 (1-5 bp), 48 (> 5 bp)	252745	161580	98.7	89.8	90.2	69	0.94	0.78
		Strelka	ins	442456	268008	161411	13037	255300	148004	95.5	84.8	92.1	67.4	255300	148004	80 (1-5 bp), 66 (> 5 bp)	76 (1-5 bp), 72 (> 5 bp)	264679	168372	98.8	96.5	91.6	69.8	0.95	0.81
			del	432376	269301	148645	14430	258316	138339	95.9	84.8	92.9	59	258316	138339	72 (1-5 bp), 33 (> 5 bp)	70 (1-5 bp), 58 (> 5 bp)	265820	154825	98.7	94.9	94.9	66.1	0.97	0.78
		Manta	ins	152945	81103	68268	3574	77155	63442	95.1	88.3	27.8	28.9	3948	8400	60 (1-5 bp), 32 (> 5 bp)	56 (1-5 bp), 54 (> 5 bp)	78946	67981	97.3	94.6	27.3	28.2	0.43	0.43
			del	495855	368746	110600	16509	217909	96106	59.1	75.6	78.4	41	150837	31003	40 (1-5 bp), 30 (> 5 bp)	58 (1-5 bp), 44 (> 5 bp)	278134	113337	75.4	89.2	99.3	48.4	0.86	0.63
	Long	NanoCaller	ins	547297	296272	226249	24776	263513	180900	89.2	72.1	95.1	82.4	32759	70125	72 (1-5 bp), 86 (> 5 bp)	64 (1-5 bp), 90 (> 5 bp)	286926	230033	96.8	91.6	99.3	95.4	0.98	0.93
			del	453591	279325	151602	22664	256318	143846	91.8	82.5	92.2	61.4	23007	30420	44 (1-5 bp), 32 (> 5 bp)	74 (1-5 bp), 72 (> 5 bp)	266260	166242	95.3	95.4	95.1	71	0.95	0.81
		PEPPER	ins	520890	287304	215378	18208	13464	33786	95.3	85.5	98.8	91	13464	33786	100 (1-5 bp), 100 (> 5 bp)	100 (1-5 bp), 100 (> 5 bp)	287304	233586	100	100	99.4	96.9	1	0.98
			del	448167	270749	160340	17078	6320	6256	97.7	96.5	95.1	73.1	6320	6256	100 (1-5 bp), 100 (> 5 bp)	100 (1-5 bp), 100 (> 5 bp)	270749	177418	100	100	96.7	75.7	0.98	0.86

*1 True positive (TP) calls that matched with the reference variants.
*2 Precision calculated with TP1 and the number of calls.
*3 Recall calculated with TP1 and the number of the reference variant.
*4 The false positive (FP) calls calculated by substitution of TP1 from the number of calls.
*5 True positive rate estimated in false positive calls by manual visual inspection of 100~200 variants randmly selected from FP calls.
*6 Corrected TP calls, which are sum of TP1 and TP estimates contained in FPs.
*7 Corrected precision calculated with TP2 and the number of calls.
*8 Corrected recall calculated with TP2 and the number of the reference variant.
*9 F-measure = 2 x Precision2 x Recall2 * 0.01 / (Precision2 + Recall2).

Supplementary Table S5 Numerical data of evaluation results for for SV calling

Sample	Read type	Algorithm	Variant type	Number of calls				TP1 *1		Precision1 (%) *2		Recall1 (%) *3		FP *4		TPR in FP (%) *5		TP2 *6		Precision2 (%) *7		Recall2 (%) *8		F-measure *9	
				Total	Non-repeat	Repeat		Non-repeat	Repeat	Non-repeat	Repeat	Non-repeat	Repeat	Non-repeat	Repeat	Non-repeat	Repeat	Non-repeat	Repeat	Non-repeat	Repeat	Non-repeat	Repeat	Non-repeat	Repeat
						STR	SegDup																		
NA12878	Short	DELLY	DEL	3637	1722	1844	71	1604	1603	93.1	83.7	72.6	17.6	118	312	6	18	1611	1659	93.6	86.6	72.9	18.2	0.82	0.3
		INSurVeyor	INS	4413	2937	1368	108	2887	1372	98.3	93	67.8	11.1	50	104	56	66	2915	1441	99.3	97.6	68.4	11.7	0.81	0.21
		GRIDSS	INS	4501	2458	1974	69	1642	1180	66.8	57.8	38.5	9.6	816	863	90	78	2376	1853	96.7	90.7	55.8	15	0.71	0.26
			DEL	4942	2062	2803	77	1731	2248	83.9	78.1	78.3	24.6	331	632	0	18	1731	2362	83.9	82	78.3	25.9	0.81	0.39
		Lumpy	INS	303	85	218	0	74	118	87.1	54.1	1.7	1	11	100	0	4	74	122	87.1	56	1.7	1	0.03	0.02
			DEL	2731	1957	725	49	1855	646	94.8	83.5	83.9	7.1	102	128	18	8	1873	656	95.7	84.8	84.8	7.2	0.9	0.13
		Manta	INS	3079	1765	1255	59	1720	1163	97.5	88.5	40.4	9.4	45	151	70	34	1752	1214	99.3	92.4	41.1	9.8	0.58	0.18
			DEL	5377	1721	3549	107	1639	2636	95.2	72.1	74.2	28.9	82	1020	32	22	1665	2860	96.7	78.2	75.3	31.3	0.85	0.45
		Wham	INS	1278	624	639	15	536	458	85.9	70	12.6	3.7	88	196	38	44	569	544	91.2	83.2	13.4	4.4	0.23	0.08
			DEL	2554	1858	635	61	1534	519	82.6	74.6	69.4	5.7	324	177	0	6	1534	530	82.6	76.1	69.4	5.8	0.75	0.11
		MOPline-S	INS	4620	3016	1471	133	2846	1393	94.4	86.8	66.8	11.3	170	211	38	42	2911	1482	96.5	92.4	68.3	12	0.8	0.21
			DEL	3413	2108	1225	80	2089	1235	99.1	94.6	94.5	13.5	19	70	16	48	2092	1269	99.2	97.2	94.7	13.9	0.97	0.24
		MOPline	INS	7225	3707	3324	194	3364	2810	90.7	79.9	78.9	22.8	343	708	50	52	3536	3178	95.4	90.3	83	25.7	0.89	0.4
			DEL	4384	2201	2075	108	2161	1814	98.2	83.1	97.8	19.9	40	369	40	38	2177	1954	98.9	89.5	98.5	21.4	0.99	0.35
	Long	cuteSV	INS	11935	3728	7916	291	3701	7984	99.3	97.3	86.9	64.9	27	223	46	78	3713	8158	99.6	99.4	87.1	66.1	0.93	0.79
			DEL	8560	1957	6368	235	1909	6195	97.5	93.8	86.4	67.9	48	408	53	70	1934	6481	98.8	98.2	87.5	71	0.93	0.82
		dysgu-LR	INS	12017	3077	8661	279	3075	8520	99.9	95.3	72.2	69.2	2	420	100	96	3077	8923	100	99.8	72.2	72.3	0.84	0.84
			DEL	10251	1946	7993	312	1940	7360	99.7	88.6	87.8	80.6	6	945	67	100	1944	8305	99.9	100	88	91	0.94	0.95
		pbsv	INS	14201	3949	9909	343	3840	9861	97.2	96.2	90.1	80.1	109	391	10	54	3851	10072	97.5	98.2	90.4	81.6	0.94	0.89
			DEL	8849	1972	6590	287	1939	6446	98.3	93.7	87.7	70.6	33	431	65	80	1960	6791	99.4	98.7	88.7	74.4	0.94	0.85
		Sniffles	INS	9376	2153	6969	254	2141	6947	99.4	96.2	50.2	56.4	12	276	100	96	2153	7212	100	99.8	50.5	58.4	0.67	0.74
			DEL	7253	1367	5579	307	1311	5400	95.9	91.7	59.3	59.2	56	486	100	100	1367	5886	100	100	61.9	64.5	0.76	0.78
		SVDSS	INS	16237	3890	11903	444	3863	11785	99.3	95.4	90.7	95.8	27	562	88	100	3887	12347	99.9	100	91.2	100	0.95	1
			DEL	10154	2019	7724	411	1967	7440	97.4	91.5	89	81.5	52	695	86	92	2012	8079	99.7	99.3	91	88.5	0.95	0.94
		SVIM	INS	14486	3704	10455	327	3689	10550	99.6	97.8	86.6	85.7	15	232	90	98	3703	10777	100	100	86.9	87.3	0.93	0.93
			DEL	9547	1994	7263	290	1974	7171	99	94.9	89.3	78.6	20	382	90	88	1992	7507	99.9	99.4	90.1	82.3	0.95	0.9
HG002	Short	DELLY	DEL	3813	1875	1877	61	1608	1552	85.8	80.1	72.5	16.2	267	386	8	4	1629	1567	86.9	80.9	73.7	17.2	0.8	0.28
		INSurVeyor	INS	4152	2624	1454	74	2588	1417	98.6	92.7	57.9	11.4	36	111	50	70	2606	1495	99.3	97.8	61.2	12.1	0.76	0.22
		GRIDSS	INS	4180	2182	1936	62	1198	1016	54.9	50.9	26.8	8.2	984	982	86	62	2044	1625	93.7	81.3	48	13.2	0.63	0.23
			DEL	5542	2537	2931	74	1727	2207	68.1	73.4	77.8	23	810	798	0	8	1727	2271	68.1	75.6	78.1	24.9	0.73	0.37
		Lumpy	INS	192	78	113	1	71	85	91	74.6	1.6	0.7	7	29	14	3	72	86	92.3	75.4	1.7	0.7	0.03	0.01
			DEL	2805	2046	712	47	1856	607	90.7	80	83.6	6.3	190	152	8	6	1871	616	91.4	81.2	84.7	6.7	0.88	0.12
		Manta	INS	2520	1265	1230	25	1241	1177	98.1	93.8	27.7	9.5	24	78	65	38	1257	1207	99.4	96.2	29.5	9.8	0.45	0.18
			DEL	4156	1711	2380	65	1600	2175	93.5	89	72.1	22.7	111	270	8	20	1609	2229	94	91.2	72.8	24.4	0.82	0.38
		Wham	INS	1071	537	525	9	465	384	86.6	71.9	10.4	3.1	72	150	70	54	515	465	95.9	87.1	12.1	3.8	0.21	0.07
			DEL	1722	1204	478	40	1067	414	88.6	79.9	48.1	4.3	137	104	4	12	1072	426	89	82.2	48.5	4.7	0.63	0.09
		MOPline-S	INS	3864	2573	1206	85	2520	1188	97.9	92	56.3	9.5	53	103	58	56	2551	1246	99.1	96.5	59.9	10.1	0.75	0.18
			DEL	3188	2056	1075	57	1982	1043	96.4	92.1	89.3	10.9	74	89	12	42	1991	1080	96.8	95.4	90.1	11.8	0.93	0.21
		MOPline	INS	7060	3561	3340	159	3265	2806	91.7	80.2	73	22.6	296	693	54	48	3425	3139	96.2	89.7	80.4	25.4	0.88	0.4
			DEL	4209	2266	1855	88	2126	1647	93.8	84.8	95.8	17.2	140	296	14	34	2146	1748	94.7	90	97.1	19.2	0.96	0.32
	Long	cuteSV	INS	12190	3780	8087	323	3746	8088	99.1	96.2	83.7	65	34	322	18	48	3752	8243	99.3	98	88.1	66.8	0.93	0.79
			DEL	8611	1970	6418	223	1940	6290	98.5	94.7	87.4	65.7	30	351	41	62	1952	6508	99.1	98	88.3	71.3	0.93	0.83
		dysgu-LR	INS	13625	3183	10128	314	3153	9729	99.1	93.2	70.5	78.2	30	713	100	96	3183	10413	100	99.7	74.7	84.3	0.86	0.91
			DEL	10834	2024	8444	366	2004	7665	99	87	90.3	80	20	1145	68	100	2018	8810	99.7	100	91.3	96.5	0.95	0.98
		pbsv	INS	14621	3984	10258	379	3863	9944	97	93.5	86.4	79.9	121	693	28	68	3897	10415	97.8	97.9	91.5	84.4	0.95	0.91
			DEL	8903	1983	6626	294	1967	6509	99.2	94.1	88.6	68	16	411	65	80	1977	6838	99.7	98.8	89.5	74.9	0.94	0.85
		Sniffles	INS	9565	2277	6986	302	2226	6822	97.8	93.6	49.8	54.8	51	466	96	94	2275	7260	99.9	99.6	53.4	58.8	0.7	0.74
			DEL	7225	1464	5402	359	1382	5340	94.4	92.7	62.3	55.8	82	421	98	98	1462	5753	99.9	99.9	66.2	63	0.8	0.77
		SVDSS	INS	15594	3833	11319	442	3759	10738	98.1	91.3	84	86.3	74	1023	92	92	3827	11679	99.8	99.3	89.8	94.6	0.95	0.97
			DEL	9471	1980	7100	391	1918	6916	96.9	92.3	86.4	72.2	62	575	74	84	1964	7399	99.2	98.8	88.9	81.1	0.94	0.89
		SVIM	INS	14987	3773	10839	375	3755	10603	99.5	94.6	83.9	85.2	18	611	65	90	3767	11153	99.8	99.5	88.4	90.3	0.94	0.95
			DEL	9546	2010	7267	269	2004	7212	99.7	95.7	90.3	75.3	6	324	17	96	2005	7523	99.8	99.8	90.7	82.4	0.95	0.9

*1 True positive (TP) calls that matched with the reference variants.

*2 Precision calculated with TP1 and the number of calls.

*3 Recall calculated with TP1 and the number of the reference variant.

*4 The false positive (FP) calls calculated by substitution of TP1 from the number of calls.

*5 True positive rate estimated in false positive calls by manual visual inspection of 100~200 variants randmly selected from FP calls.

*6 Corrected TP calls, which are sum of TP1 and TP estimates contained in FPs.

*7 Corrected precision calculated with TP2 and the number of calls.

*8 Corrected recall calculated with TP2 and the number of the reference variant.

*9 F-measure = 2 x Precision2 x Recall2 * 0.01 / (Precision2 + Recall2).

Supplementary Table S6 Optimal variant detection algorithms

Variant type	Read type	Algorithm	Comment
SNV	Short	DeepVariant	The top performance in short read-based algorithms. Low recall in repetitive regions.
		GATK4	Second best. Low recall in repetitive regions.
	Long	NanoCaller	The top for SNV calling. Nearly 100% precision and recall.
		PEPPER	Slightly lower recall in repetitive regions.
Indel	Short	DeepVariant	The top for short read-based SV calling.
		GATK4	Second best. High recall in larger size ranges of insertion
		Strelka	Second best.
	Long	PEPPER	The top for indel calling. Nearly 100% precision and recall for insertion calling.
SV (INS/DEL)	Short	MOPline	The top for short read-based SV calling.
		Manta	High precision particularly for DEL calling
		INSurVeyor	High precision and recall for INS calling
	Long	SVDSS	The top for SV calling.
		SVIM	Second best. Slightly low recall for INS calling.
		pbsv	Third best. Slightly low recall for INS calling.
		dysgu	High recall for DEL calling in repetitive regions.
