# Supplementary material for: Comparative evaluation of SNVs, indels, and structural variations detected with short- and long-read sequencing data
Source: Hum Genome Var. 2024 Apr 17;11:18. doi: 10.1038/s41439-024-00276-x (PMC11024196; doi:10.1038/s41439-024-00276-x)
Supplement: Supplementary file 2 — Supplementary Note [file 41439_2024_276_MOESM2_ESM.pdf]

Supplementary Note:

Variant calling procedures

The commands, options, and filtering conditions used for the algorithms are as follows: (input bam, reference fasta, and output prefix are denoted as ${bam}, ${ref}, and ${out}, respectively.)

DeepVariant (v1.3.0)

Command: singularity exec --bind ${dir} deepvariant_1.3.0.sif /opt/deepvariant/bin/run_deepvariant --model_type WGS --reads ${bam} --ref ${ref} --output_vcf ${out}.vcf.gz --num_shards 8

${dir} are the directories to be mounted within the singularity container and ${work_dir} is the working directory. Variants that were indicated as 'RefCall' in the FILTER field of the output vcf were excluded.

GATK4 (v4.1.2)

BaseRecalibrator command: gatk BaseRecalibrator -I ${bam} -R ${ref} --known-sites ${known_sites} -O ${out}.recal.table

ApplyBQSR command: gatk ApplyBQSR -I ${bam} -R ${ref} --bqsr-recal-file ${out}.recal.table -O ${out}.recal.bam

HaplotypeCaller command: gatk HaplotypeCaller -ERC GVCF -I ${out}.recal.bam -R ${ref} -G StandardAnnotation -G AS_StandardAnnotation -G StandardHCAnnotation -O ${out}.HC.g.vcf.gz

The known variant data sets (${known_sites}) include dbsnp_138.b37.vcf, 1000G_phase1.snps.high_confidence.b37.vcf, 1000G_phase1.indels.b37.vcf, Mills_and_1000G_gold_standard.indels.b37.vcf, and 1000G_phase3_v4_20130502.sites.vcf, which were obtain from ftp://ftp.broadinstitute.org//bundle/b37/. Among the variants in the output gvcf, variants with < 2 AD, < 2.0 QD (quality / DP), < -8.0 ReadPosRankSum for SNVs (< -20 for indel), and < -12.5 MQRankSum (SNV only) were excluded.

Lofreq (v2.1.5)

Preprocee command: lofreq indelqual -f ${ref} --dindel -o ${out}.addQual.bam ${bam}

Call command: lofreq call-parallel -f ${ref} --pp-threads 6 -o ${out}.vcf --call-indels ${out}.addQual.bam

Strelka (v2.9.10)

1st command: configureStrelkaGermlineWorkflow.py --bam ${bam} --referenceFasta ${ref} --runDir ./

2nd command: ./runWorkflow.py -m local -j 6 -g 60

Platypus (v0.8.1)

Command: python Platypus.py callVariants --genSNPs=0 --minReads=3 --maxSize=1500 --minMapQual=0 --minBaseQual=10 --nCPU=$cores --refFile=${ref} --bamFiles=${bam} --output=${out}.vcf

NanoCaller (v3.4.1)

Command: singularity run --bind ${dir} nanocaller_3.4.1.sif NanoCaller --bam ${bam} --ref ${ref} --output ${work_dir} --cpu 6 --preset ccs

PEPPER-Mergin-DeepVariant (r0.8)

Command: singularity exec --bind ${dir} pepper_deepvariant_r0.8.sif run_pepper_margin_deepvariant call_variant -b ${bam} -f ${ref} -o ${work_dir} -p ${out} -t 6 –hifi

Variants that were indicated as 'RefCall' in the FILTER field of the output vcf were excluded.

Manta (v1.6.0)

1st command: configManta.py --bam ${bam} --referenceFasta ${ref} --runDir ./

2nd command: ./runWorkflow.py -m local -j 6 -g 100

For short indel calling, configManta.py in the bin_short_indel directory was used to run. INV, BND, and non-PATH variants were excluded.

DELLY (v1.1.8)

Command: delly_v1.1.8_linux_x86_64bit call -g ${ref} -o ${out}.bcf ${bam}

The output bcf file was converted to vcf format, and Non-PASS DEL and DUP calls were excluded.

GRIDSS (v2.13.2)

Command: gridss --reference $ref_base --output ${out}.vcf.gz --assembly ${out}.gridss.assembly.bam --jar gridss-2.13.2-gridss-jar-with-dependencies.jar --jvmheap 40g --blacklist ${black_list_bed} --threads 6 --keepTempFiles ${bam}

SVs with LOW_QUAL or NO_ASSEMBLY in the FILTER field of the output vcf or in the duplicated positions were excluded.

INSurVeyour (v1.1.1)

Command: singularity run --bind ${dir} insurveyor.sif --threads 6 --min-insertion-size ${min_size} ${bam} ./ ${ref}

The min_size was specified to 1 for indel calling and 50 for SV calling.

Lumpy (v0.3.1)

Pre-processing of the input bam was conducted according to the description on the github site (https://github.com/arq5x/lumpy-sv).

Run command: lumpy -mw 4 -tt 0.0 -pe bam_file:${out}.discordant.sort.bam,histo_file:${out}.histo,mean:500,stdev:100,read_length:150,min_non_overlap:150,discordant_z:4,back_distance:20,weight:1,id:1,min_mapping_threshold:20 -sr bam_file:${out}.sr.sort.bam,back_distance:20,weight:1,id:2,min_mapping_threshold:20 > ${out}.pesr.bedpe

SV calls ending with '0:0:0' at the 10th or 11th column of the output file and BND calls were excluded.

Wham (v1.8.0)

Command: whamg -f ${bam} -a ${ref} -c 1,2,3,4,5,6,7,8,9,10,11,12,13,14,15,16,17,18,19,20,21,22,X -x 6 > ${out}.vcf

SVs with < 0.2 weight value for each type in the output vcf were excluded.

MOPline (v1.8.2)

SVs were called using MOPline-7t, which selectively integrates overlap calls between the call sets from seven short read-based algorithms (CNVnator, GRIDSS, inGAP-sv, Manta, MATCHCLIP, MELT, and Wham). The run with a single sample (MOPline-S) was performed the steps-0 to -3, and -6 according to the manual described in the github site (https://github.com/stat-lab/MOPline). The run with multiple samples was performed all the steps of MOPline using 99 1KGP CEU WGS data for NA12878 or using the 1KGP CEU data plus the HG002 data for HG002. For the run with multiple samples, SVs corresponding to NA12787 or HG002 were extracted from the filtered output joint-call/SMC vcf files.

cuteSV (v1.0.13)

Command: cuteSV ${bam} ${ref} ${out}.vcf ./ --threads 6 --min_size [3 or 50] --max_cluster_bias_INS 1000 --diff_ratio_merging_INS 0.9 --max_cluster_bias_DEL 1000 --min_support 2 –genotype

Non-PASS variants and BND variants were excluded.

Dysgu (v1.3.10)

Short read command: dysgu run -p6 -o ${out}.vcf --min-size 3 --min-support 2 -x ${ref} ./tmp ${bam}

Long read command: dysgu call --mode pacbio -p6 -o ${out}.vcf --min-size 50 --min-support 2 -x ${ref} ./tmp ${bam}

Variants with < 3 supporting reads, which were indicated by SU tags in the output vcf, were excluded.

pbsv (v2.8.0)

Discover command: pbsv discover -s NA -l 50 --hifi ${bam} ${out}.svsig.gz

Call command: pbsv call -j 6 -t DEL,INS,DUP -m [3 or 50] -A 2 -O 2 ${ref} ${out}.svsig.gz ${out}.pbsv.vcf

Sniffles (v2.0.7)

Command: sniffles -i ${bam} --minsvlen [3 or 50] --long-ins-length 10000 --long-del-length 100000 -t 6 -v ${out}.vcf

Variants with < 2 supporting reads, as indicated at the FORMAT field of the output vcf, were excluded.

SVDSS (v1.0.5)

Index command: SVDSS index –reference ${ref} --index ${ref}.index

Smooth command: SVDSS smooth --reference ${ref} --bam ${bam} --workdir ./ --threads 6

Search command: SVDSS search --index ${ref}.index --bam smoothed.selective.bam --workdir ./ --threads 6 --assemble

Call command: SVDSS call --reference ${ref} --bam smoothed.selective.bam --workdir ./ --threads 6 --min-sv-length 50 --min-cluster-weight 2 --batches 93

SVIM (v2.0.0)

Command: svim alignment --min_sv_size [3 or 50] --max_sv_size 1000000 . ${bam} ${ref}

Variants with 'hom_ref' in the INFO field of the output vcf were excluded.

For SVs/indels called by long read-based algorithms, overlapped variants of the same type at the same or nearly the same position (≦ 50 bp distance for ins/INS and ≧ 50% reciprocal overlap for DEL/DUP) were excluded from the analysis when the size ratio of the overlapped variants was between 0.67 and 1.5 because of the possibility of making

false duplicate calls.
